# “Energetic” Cancer Stem Cells (e-CSCs): A New Hyper-Metabolic and Proliferative Tumor Cell Phenotype, Driven by Mitochondrial Energy

**DOI:** 10.3389/fonc.2018.00677

**Published:** 2019-02-05

**Authors:** Marco Fiorillo, Federica Sotgia, Michael P. Lisanti

**Affiliations:** ^1^Biomedical Research Centre (BRC), Translational Medicine, School of Environment and Life Sciences, University of Salford, Manchester, United Kingdom; ^2^The Department of Pharmacy, Health and Nutritional Sciences, The University of Calabria, Cosenza, Italy

**Keywords:** cancer stem-like cells (CSCs), metabolism, mitochondrial OXPHOS, glycolysis, Diphenyleneiodonium (DPI), Ribociclib

## Abstract

Here, we provide the necessary evidence that mitochondrial metabolism drives the anchorage-independent proliferation of CSCs. Two human breast cancer cell lines, MCF7 [ER(+)] and MDA-MB-468 (triple-negative), were used as model systems. To directly address the issue of metabolic heterogeneity in cancer, we purified a new distinct sub-population of CSCs, based solely on their energetic profile. We propose the term “energetic” cancer stem cells (e-CSCs), to better describe this novel cellular phenotype. In a single step, we first isolated an auto-fluorescent cell sub-population, based on their high flavin-content, using flow-cytometry. Then, these cells were further subjected to a detailed phenotypic characterization. More specifically, e-CSCs were more glycolytic, with higher mitochondrial mass and showed significantly elevated oxidative metabolism. e-CSCs also demonstrated an increased capacity to undergo cell cycle progression, as well as enhanced anchorage-independent growth and ALDH-positivity. Most importantly, these e-CSCs could be effectively targeted by treatments with either (i) OXPHOS inhibitors (DPI) or (ii) a CDK4/6 inhibitor (Ribociclib). Finally, we were able to distinguish two distinct phenotypic sub-types of e-CSCs, depending on whether they were grown as 2D-monolayers or as 3D-spheroids. Remarkably, under 3D anchorage-independent growth conditions, e-CSCs were strictly dependent on oxidative mitochondrial metabolism. Unbiased proteomics analysis demonstrated the up-regulation of gene products specifically related to the anti-oxidant response, mitochondrial energy production, and mitochondrial biogenesis. Therefore, mitochondrial inhibitors should be further developed as promising anti-cancer agents, to directly target and eliminate the “fittest” e-CSCs. Our results have important implications for using e-CSCs, especially those derived from 3D-spheroids, (i) in tumor tissue bio-banking and (ii) as a new cellular platform for drug development.

## Introduction

Cancer stem cells (CSCs) are tumor-initiating cells (TICs) which are resistant to conventional cancer therapies, such as chemo-therapy and radiation treatment. As a consequence, CSCs are responsible for (i) tumor recurrence and (ii) distant metastasis, driving treatment failure, and poor clinical outcomes in cancer patients (1, 2). Therefore, innovative approaches are necessary to understand how to tackle the problem of CSCs. Mechanistically, this may be related to the ability of CSCs to survive and thrive under harsh conditions and different micro-environments (1–4). Since CSCs are an especially small sub-set of the tumor cell population, their metabolic and phenotypic properties have remained largely uncharacterized, until recently.

Moreover, CSCs are strikingly resilient and highly resistant to cellular stress, which allows them to undergo anchorage-independent growth, especially under conditions of low-attachment (1, 2). As a consequence, they form 3D spheroids, which retain the properties of CSCs and stem cell progenitors. In contrast, when subjected to growth in suspension, most “bulk” cancer cells die, via anoikis—a specialized type of apoptosis. As such, the clonal propagation of a single CSC results in the production of a 3D spheroid and does not involve the self-aggregation of cancer cells. Therefore, 3D spheroid formation is a functional read-out for stemness in epithelial cancer cells and allows one to enrich for a population of epithelioid cells with a stem-like phenotype. These 3D spheroids are also known as mammospheres when they are prepared using breast cancer cells, such as MCF7, among others.

Previously, we generated 3D spheroids from 2 distinct ER(+) cells lines (MCF7 and T47D) and scaled-up their preparation so that they could be subjected to unbiased label-free proteomics analysis (5). This approach was taken to begin to decipher the phenotypic behavior of CSCs at a molecular level. 3D spheroids were directly compared with monolayers of these cell lines and processed in parallel. This allowed us to determine the proteomic features that are characteristic of the CSC phenotype in 3D spheroids, relative to monolayers. Based on this molecular analysis, we observed that mammospheres are significantly enriched in mitochondrial proteins. These mitochondrial-related proteins included molecules involved in beta-oxidation and ketone metabolism/re-utilization, mitochondrial biogenesis, electron transport, ADP/ATP exchange/transport, CoQ synthesis and ROS production, as well as the suppression of mitophagy. As such, increased mitochondrial protein synthesis or decreased mitophagy could allow the accumulation of mitochondrial mass in CSCs. As a consequence, we proposed that CSCs might become resistant to conventional therapies, by “boosting” ATP production using elevated mitochondrial OXPHOS metabolism.

Consistent with this view, a variety of mitochondrial inhibitors successfully blocked 3D tumor sphere formation, including (i) FDA-approved antibiotics (doxycycline, tigecycline, azithromycin, pyrvinium pamoate, atovaquone, bedaquiline), (ii) natural compounds (actinonin, CAPE, berberine, brutieridin, and melitidin), as well as (iii) experimental compounds [oligomycin and AR- C155858 (an MCT1/2 inhibitor)] (6).

Taken together, all of these findings prompted us to employ mitochondrial mass as a new metabolic biomarker to purify CSCs. Using this overall approach, we observed that it was possible to significantly enrich CSC activity using only MitoTracker, as a single marker for both ER(+) (MCF7) and ER(–) (MDA-MB-231) breast cancer cell lines. Remarkably, the MitoTracker-high cells were chemo-resistant to Paclitaxel, exhibiting resistance to the Paclitaxel-induced DNA-damage response (7).

One emerging idea is that there is diverse metabolic heterogeneity in the CSC population (3, 4). To test this hypothesis, here we used a flow-cytometry approach to metabolically fractionate the cancer cell population into “low-energy” and “high-energy” cell sub-populations. For this purpose, we used auto-fluorescence as an endogenous marker of their energetic state. In this context, we focused on auto-fluorescence attributed to the endogenous flavin-containing metabolites, such as FAD, FMN and riboflavin (Vitamin B2) (8–16). Most importantly, we investigated if their growth in a 2D or 3D micro-environment affected their metabolic rate and stem-like properties.

Our current results provide novel evidence for the existence of an “energetic” CSC phenotype, which may represent the “fittest” CSCs. Remarkably, these e-CSCs are both (i) metabolically-active and (ii) hyper-proliferative, as well as (iii) critically-dependent on a 3D micro-environment.

## Materials and Methods

### Breast Cancer Cell Models and Other Reagents

Human breast cancer cell lines, MCF7 [ER(+)] and MDA-MB-468 (triple-negative), were obtained commercially from the ATCC. Both cell lines were maintained in Dulbecco's Modified Eagle Medium (DMEM; GIBCO), supplemented with 10% FBS, 1% Glutamax, and 1% Penicillin-Streptomycin. All cell lines were maintained at 37°C in 5% CO_2_. Diphenyleneiodonium chloride (DPI) and Ribociclib were purchased from Sigma-Aldrich, Inc.

### Cell Sorting: Flow-Cytometry and Collection of Auto-Fluorescent Cells

MCF7 and MDA-MB-468 cells were first grown either as a 2D-monolayer or as 3D-spheroids (8, 14, 16, 17). Then, they were collected and dissociated into a single-cell suspension, prior to analysis or sorting by flow-cytometry with the SONY SH800 Cell Sorter. Briefly, auto-fluorescent cells were excited with a 488 nm blue laser and selected at the intersection with filters 525/50 and 585/30. The **“**Low” and “High” auto-fluorescent cell sub-populations were selected by gating, within the auto-fluorescence signal. Only cells with the least (bottom 5%) or the most (top 5%) auto-fluorescence signal were collected. The cells outside the gates were discarded during sorting, due to the gate settings. However, such settings are required, to ensure high-purity during sorting. To better characterize the auto-fluorescent cell sub-populations, the following flow-cytometry markers were used: ALDEFLUOR-assay (StemCell technologies, Durham, NC, USA); and MitoTracker Deep Red (Thermo Fisher Scientific). Hoescht (Thermo Fisher Scientific) was used for cell cycle analysis. Data were analyzed with FlowJo 10.1 software.

### Preparing Cells for Auto-Fluorescent Cell Sorting by Flow-Cytometry

We used the following protocol to acquire and sort auto-fluorescent cells from 2D-monolayers or 3D-spheroid cell suspensions. For 2D-monolayers, we seeded MCF7 and MDA-MB-468 in a 225 cm^2^ flask and when ~70% confluence was reached, 5 ml of 0.025% trypsin was added to the flasks and incubated at 37°C for 5 min. After that the cells were re-suspended in media and centrifuged at 300 g for 5 min. After centrifugation, the cell pellets were adjusted to a concentration of 10^6^ cells/ml in in PBS Ca/Mg for acquisition or in sorting buffer [1× PBS containing 3% (v/v) FBS and 2 mM EDTA] for FACS.

For 3D-spheroid suspensions, after 5 days of growth under low-attachment condition, the spheres were collected from six 225 cm^2^ flasks pre-coated with poly-HEMA and gently centrifuged at 100 g for 5 min. After centrifugation, we added 1 ml of 0.025% of trypsin to the “sphere-pellet” and incubated them at 37°C for 5 min. Using a 25-gauge needle, we passed the sphere-suspension through the syringe 4 times. The sphere suspension was then centrifuged again at 100 g for 5 min, and the sphere-pellet was re-suspended in (i) PBS Ca/Mg for acquisition or (ii) in sorting buffer [1× PBS containing 3% (v/v) FBS and 2 mM EDTA] for FACS and we “syringed” the suspension again 4 times.

After creating these single-cell suspensions, we subjected them to standard flow-cytometry (using the SONY SH800 Cell Sorter) to isolate the auto-fluorescent cell sub-populations, as indicated above. Examples of flow-cytometry plots are included in the figures, and the gating strategy is shown.

### Mammosphere Formation Assay (for Generating 3D-Spheroids)

A single-cell suspension was prepared using enzymatic, and manual disaggregation (25 gauge needle) (18). Then, cells were plated at a density of 500 cells/cm^2^ in mammosphere medium (DMEM-F12 + B27 + 20 ng/ml EGF + PenStrep) under non-adherent conditions, in culture dishes pre-coated with (2-hydroxyethylmethacrylate) (poly-HEMA, Sigma, #P3932), called “mammosphere plates.” Cells were grown for 5 days and maintained in a humidified incubator at 37°C. After 5 days of culture, 3D-spheres >50 μm were counted using an eye piece (“graticule”), and the percentage of cells plated which formed spheres was calculated and is referred to as percent mammosphere formation, and was normalized to one (1 = 100% MFE). Mammosphere formation efficiency was analyzed in both the “low” and “high” sub-populations of auto-fluorescent cells, generated from either 2D-monolayers (M-L vs. M-H) or 3D-spheroids (S-L vs. S-H). All mammosphere experiments were performed in triplicate, at least 3 times independently.

### ALDEFLUOR Assay

The level of ALDH activity was assessed, by using the fluorescent reagent ALDEFLUOR. The ALDEFLUOR kit (StemCell technologies, Durham, NC, USA) was used to detect the cell sub-populations with various amounts of ALDH enzymatic activity by FACS (Attune NxT Flow Cytometer). Briefly, 1 × 10^5^ cells were incubated in 1 ml ALDEFLUOR assay buffer containing ALDH substrate (5 μl/ml) for 40 min at 37°C. In each experiment a sample of cells was stained under identical conditions with 30 μM of diethylaminobenzaldehyde (DEAB), a specific ALDH inhibitor, as a negative control The ALDH-positive population was established, according to the manufacturer's instructions and was evaluated using 50,000 cells. All the ALDH experiments were performed three times independently.

### Seahorse XFe96 Metabolic Flux Analysis

Real-time oxygen consumption rates (OCRs) and extracellular acidification rates (ECAR) rates were determined using the Seahorse Extracellular Flux (XFe96) analyzer (Seahorse Bioscience, USA) (19–21). Briefly, 2 × 10^4^ cells per well were seeded into XFe96 well cell culture plates after sorting, and incubated for 12 h to allow cell attachment. After 12 h of incubation, cells were washed in pre-warmed XF assay media (or for OCR measurement, XF assay media supplemented with 10 mM glucose, 1 mM Pyruvate, 2 mM L-glutamine, and adjusted at 7.4 pH). Cells were then maintained in 175 μL/well of XF assay media at 37°C, in a non-CO_2_ incubator for 1 h. During the incubation time, we loaded 25 μL of 80 mM glucose, 9 μM oligomycin, and 1M 2-deoxyglucose (for ECAR measurement) or 10 μM oligomycin, 9 μM FCCP, 10 μM rotenone, 10 μM antimycin A (for OCR measurement), in XF assay media into the injection ports in the XFe96 sensor cartridge (20, 21). Measurements were normalized by protein content (SRB assay) and Hoechst 33342 content. Data sets were analyzed using XFe96 software and GraphPad Prism software, using one-way ANOVA and Student's *t*-test calculations. All experiments were performed in quintuplicate, three times independently.

### Vital Mitochondrial Staining

Cells were trypsinized and re-suspended into a 1 × 10^6^ cell/ml solution in PBS. MitoTracker Deep-Red (10 nM; Thermo Fisher Scientific) was added for 30 min at 37°C before centrifugation and re-suspension in PBS Ca/Mg for FACS analysis (ATTUNE NxT) or Cell Sorting (SONY SH 800). All subsequent steps were performed in the dark. Data analysis was performed using FlowJo software.

### Cell Cycle Analysis

We performed cell-cycle analysis on the auto-fluorescent cell sub-populations, by FACS analysis using the SONY Cell Sorter. Briefly, after trypsinization, the re-suspended cells were incubated with 10 ng/ml of Hoescht solution (Thermo Fisher Scientific) for 40 min at 37°C under dark conditions. Following a 40 min period, the cells were washed and re-suspended in PBS Ca/Mg for acquisition or in sorting buffer [1× PBS containing 3% (v/v) FBS and 2 mM EDTA] for FACS. We analyzed 50,000 events per condition Gated cells were manually-categorized into cell-cycle stages.

### Statistical Analysis

All analyses were performed with GraphPad Prism 6. Data were represented as mean ± SD (or ± SEM where indicated). All experiments were conducted at least 3 times independently, with >3 technical replicates for each experimental condition tested (unless stated otherwise, e.g., when representative data is shown). Statistically significant differences were determined using the Student's *t*-test or the analysis of variance (ANOVA) test. For the comparison among multiple groups, one-way ANOVA were used to determine statistical significance. *P* ≤ 0.05 was considered significant and all statistical tests were two-sided.

### Proteomics Analysis

Label-free unbiased proteomics and Ingenuity pathway analysis (IPA) were carried out, essentially as previously described, using standard protocols, with relatively minor modifications (5, 22–25).

#### Ingenuity Pathway Analysis (IPA)

Unbiased interrogation and analysis of our proteomic data sets was carried out by employing a bioinformatics platform, known as IPA (Ingenuity systems, http://www.ingenuity.com). IPA assists with data interpretation, via the grouping of differentially expressed genes or proteins into known functions and pathways. Pathways with a z score of > +2 were considered as significantly activated, while pathways with a z score of <-2 were considered as significantly inhibited.

#### Clinical Relevance of e-CSC Marker Proteins

To validate the clinical relevance of our findings, we first assessed whether the e-CSC targets that we identified in MCF7 cells were also transcriptionally upregulated in human breast cancer cells *in vivo*. For this purpose, we employed a published clinical data set of *N* = 28 breast cancer patients in which their tumor samples were subjected to laser-capture micro-dissection (5, 26), to physically separate epithelial cancer cells from their adjacent tumor stroma.

#### Kaplan-Meier (K-M) Analyses

To perform K-M analysis on mRNA transcripts, we used an open-access online survival analysis tool to interrogate publically available microarray data from up to 3,455 breast cancer patients. This allowed us to determine their prognostic value (27). For this purpose, we primarily analyzed data from ER(+) patients that were LN(+) at diagnosis and were of the luminal A sub-type, that were primarily treated with tamoxifen and not other chemotherapy (*N* = 150 patients). In this group, 100% the patients received some form of hormonal therapy and ~95% of them received tamoxifen. Biased and outlier array data were excluded from the analysis. This allowed us to identify metabolic gene transcripts, with significant prognostic value. Hazard-ratios were calculated, at the best auto-selected cut-off, and *p*-values were calculated using the log-rank test and plotted in *R*.

K-M curves were also generated online using the K-M-plotter (as high-resolution TIFF files), using univariate analysis: http://kmplot.com/analysis/index.php?p=service&cancer=breast.

This allowed us to directly perform *in silico* validation of these metabolic biomarker candidates. The 2017 version of the database was utilized for all these analyses, while virtually identical results were also obtained with the 2014 and 2012 versions.

## Results

### Dissecting Metabolic Heterogeneity in CSCs

Here, we used two human breast cancer cell lines (i.e., MCF7 and MDA-MB-468) as model systems, to dissect the role of metabolic heterogeneity in tumorigenesis. Results with MCF7 cells are shown in the main text **Figures 4–11**, Tables 1–**3** and Tables S1–S6, while results with MDA-MB-468 cells are included in Figures S1–S3. MCF7 cells are ER(+), while MDA-MB-468 cells are triple-negative. Quantitatively similar results were obtained with both model cell lines.

**Table 1:** MCF7-derived e-CSCs cells demonstrate increased cell cycle progression.

**CC-Phase (%)**	**2D-Monolayers (M)**		**3D-Spheroids (S)**	
	**M-L**	**M-H**	**S-L**	**S-H**
G0/G1	81.25	53.23	61.50	37.32
S-phase	3.92	11.43	6.72	10.60
G2/M	8.53	21.23	11.72	32.43
Polyploid	3.71	10.74	9.03	17.13

*Averages are shown from 4 independent experiments. M-L, monolayer-low; M-H, monolayer-high; S-L, spheroid-low; S-H, spheroid-high. See Figure 5A for specific SD*.

**Figure 1:**
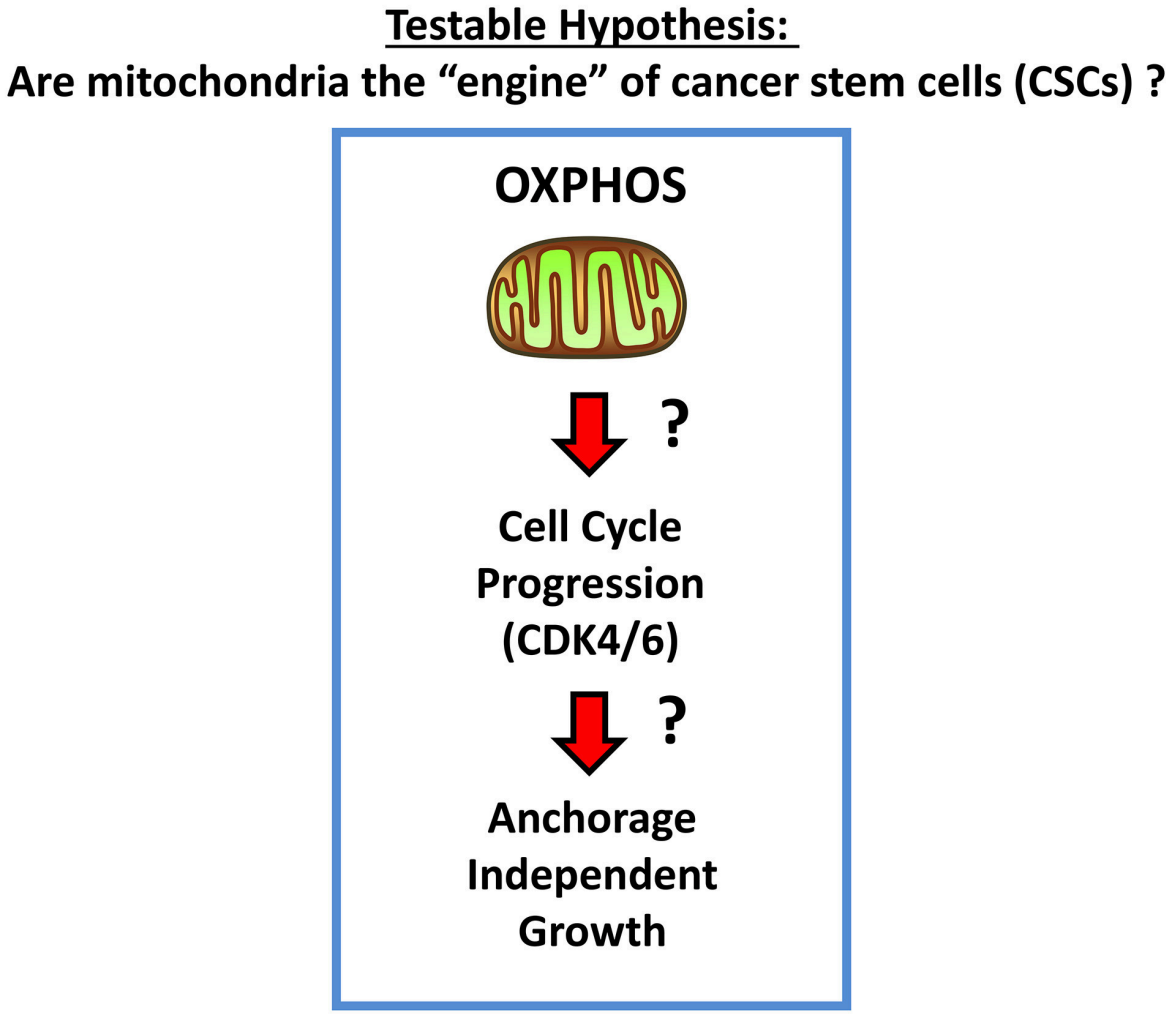
Mitochondria and CSCs. Here, we propose the testable hypothesis that mitochondria are the “engines” that drive cell-cycle progression and proliferation in CSCs, especially under anchorage-independent growth conditions.

**Figure 2:**
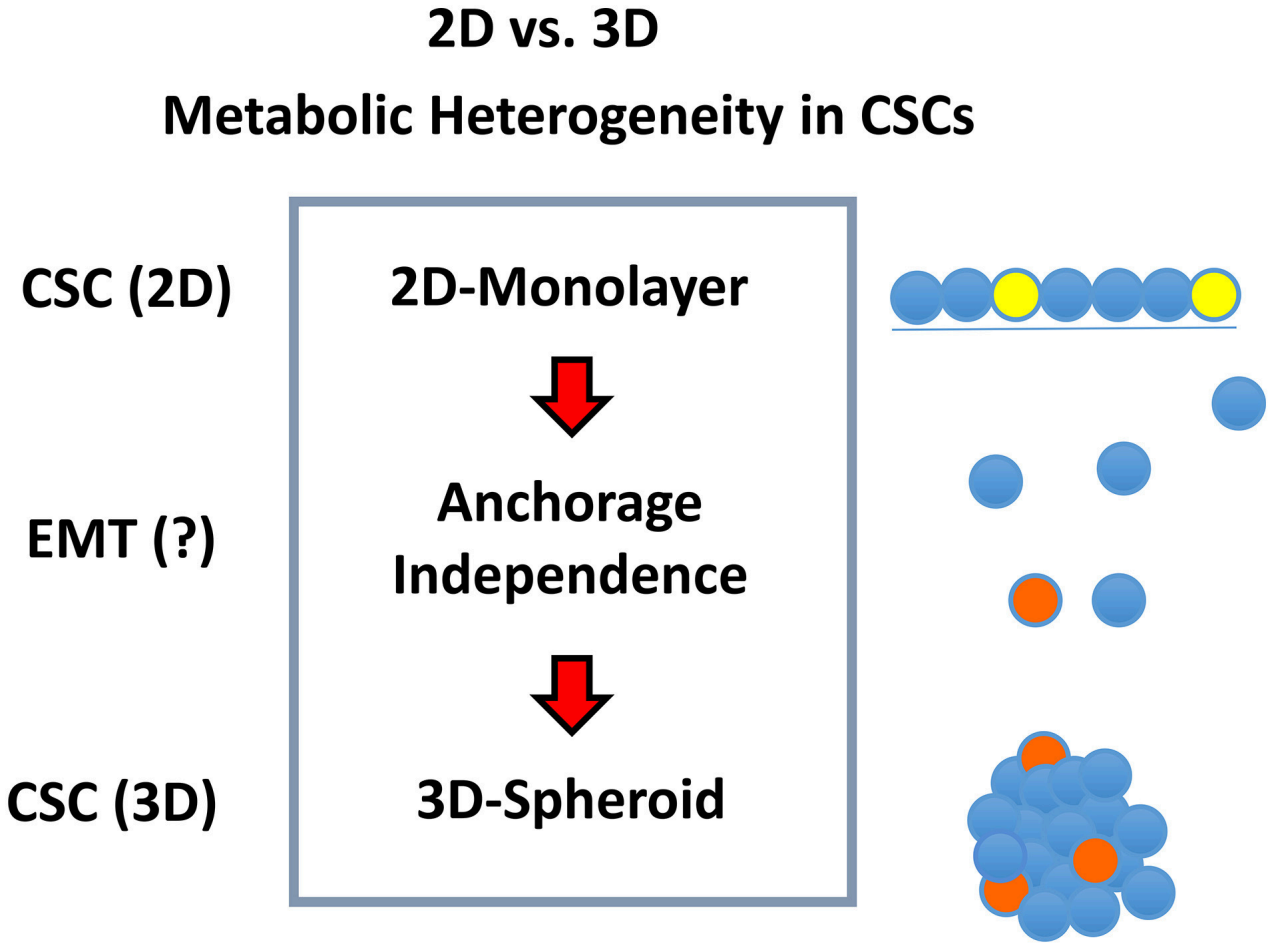
Metabolic heterogeneity in CSCs. We hypothesized that different sub-types or sub-classes of CSCs may exist and that they may differ in their energetic phenotype, based on whether they are physically grown as either 2D-monolayers or 3D-spheroids. We speculated that the transition from a 2D environment to a 3D format would involve an epithelial mesenchymal transition (EMT). Due to the stress of the transition from 2D to 3D growth, it could require additional energy production, possibly afforded by mitochondrial metabolism.

**Figure 3:**
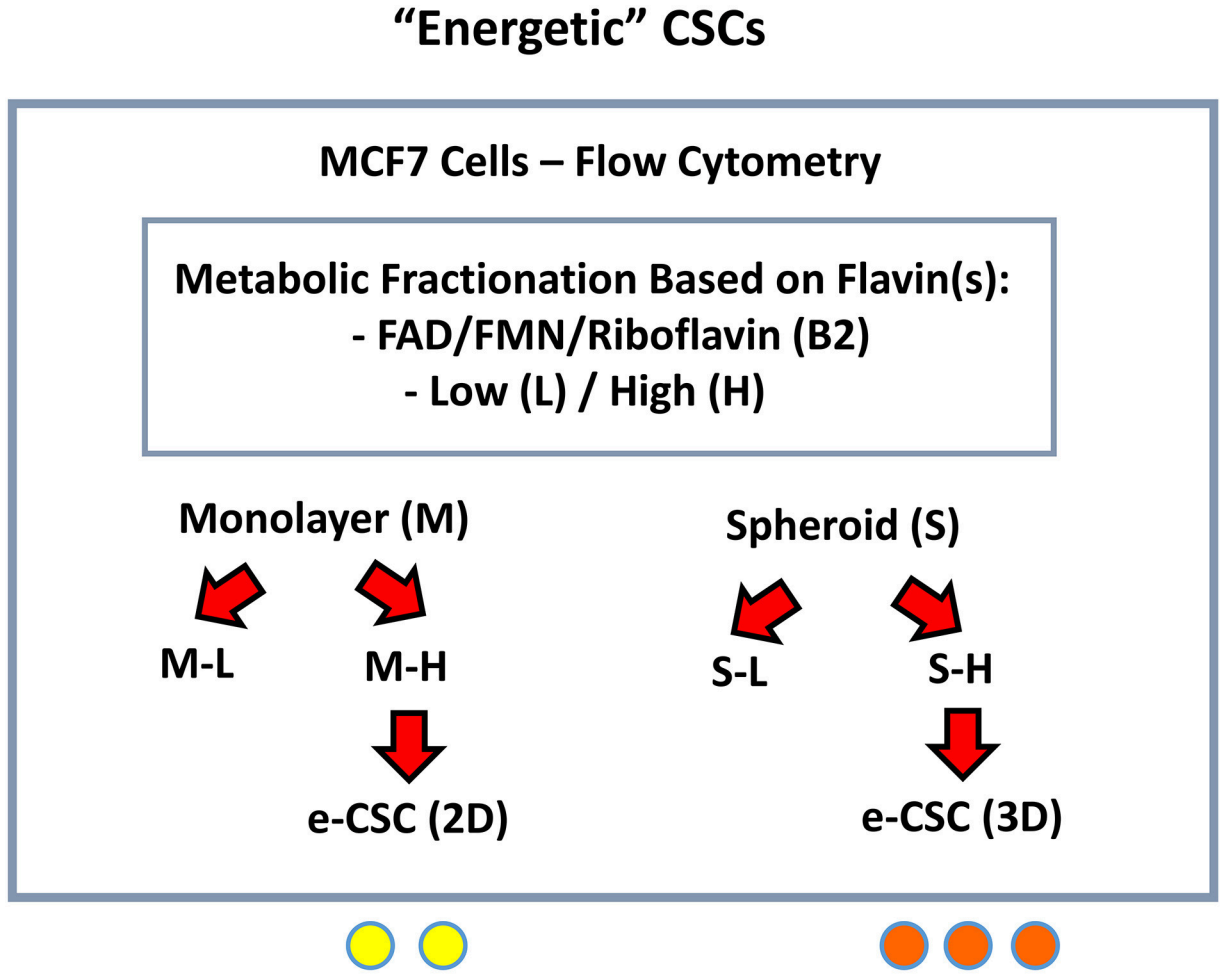
Detecting and purifying “energetic” CSCs (e-CSCs). In order to isolate CSCs, single cell suspensions of MCF7 cells were first subjected to metabolic fractionation by flow cytometry, based on the endogenous auto-fluorescence (AF) of FAD/FMN, high-energy metabolites. The high (H) and low (L) sub-populations of AF cells were then collected from MCF7 cells grown either as (i) 2D-monolayers or (ii) 3D-spheroids. These “high-energy” AF(+) cells were then designated as: e-CSCs (2D) and e-CSCs (3D).

We hypothesized that mitochondria may function as the metabolic “engines” to drive cellular hyper-proliferation and, ultimately, anchorage-independent growth, leading to tumor recurrence and metastasis (Figure 1). Moreover, we speculated that at least two different sub-populations of CSCs may exist, depending on whether the cells are grown as 2D-monolayers or as 3D-spheroids (Figure 2). To investigate this hypothesis, we used cell auto-fluorescence as an endogenous marker of cellular energy metabolism, which directly reflects their content of flavin-containing compounds [FAD, FMN, and riboflavin (Vitamin B2)], which are all high-energy cell metabolites (Figure 3).

As a consequence, 2D-monolayers and 3D-spheroids were first collected and used to prepare single-cell suspensions. These suspensions were then subjected to flow-cytometry to isolate cells based on their auto-fluorescent properties. Briefly, the **“**Low-(L)” and “High-(H)” auto-fluorescent cell sub-populations were selected by gating, within the auto-fluorescence signal. Only cells with the least (bottom 5%) or the most (top 5%) auto-fluorescent signal were collected.

Both the “Low” and “High” sub-populations of auto-fluorescent cells, generated from either 2D-monolayers (M-L vs. M-H) or 3D-spheroids (S-L vs. S-H) were then subjected to a detailed phenotypic characterization (Figure 3). The M-H (“monolayer-high”) and S-H (“spheroid-high”) cell sub-populations were predicted to be the most energetic, based on their high (H) flavin-content.

**Figure 4:**
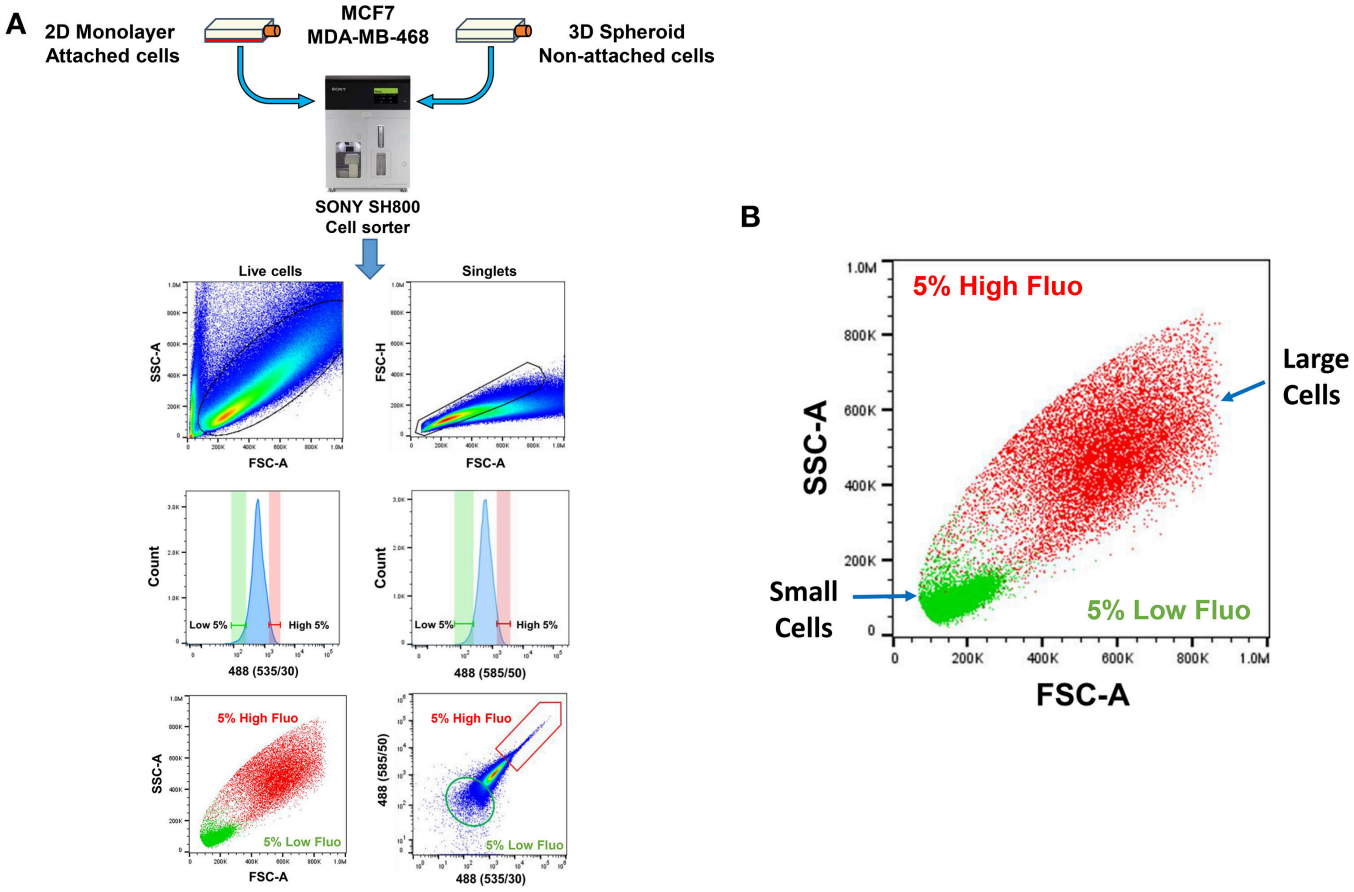
Technical scheme for isolating e-CSCs, via flow-cytometry. **(A)** See “Experimental Procedures” for a more detailed explanation. Briefly, single-cell suspensions were prepared from 2D-monolayers or 3D-spheroid cell cultures. Then, these single-cell suspensions were subjected to flow-cytometry, using the SONY SH800 cell sorter. This allowed us to select the flavin-specific auto-fluorescent (AF) cell population by gating. First, only live cells were selected; then, only singlet cells were selected, by using the forward- and side-scatter parameters. Ultimately, the “AF-high” (top 5%) and the “AF-low” (bottom 5%) sub-populations were selected, by plotting the signal from the 488(535/30) nm laser vs. the 488 (585/50) nm laser. **(B)** Plotting forward scatter vs. side scatter gives an estimate of relative cell size. Note that AF-high cells are considerably larger than AF-low cells. This may also reflect their increased metabolic activity.

A more detailed technical version of this enrichment work-flow is briefly summarized in Figure 4A, as accomplished via flow-cytometry. Based on the forward- and side-scatter analysis of single cells, highly auto-fluorescent cells (shown in RED) are clearly larger in size, than cells with low auto-fluorescence (shown in GREEN) (Figure 4B).

### Characterization of the e-CSC Phenotype: Proliferation, “Stemness” and Bioenergetics

#### Proliferation

**Figure 5:**
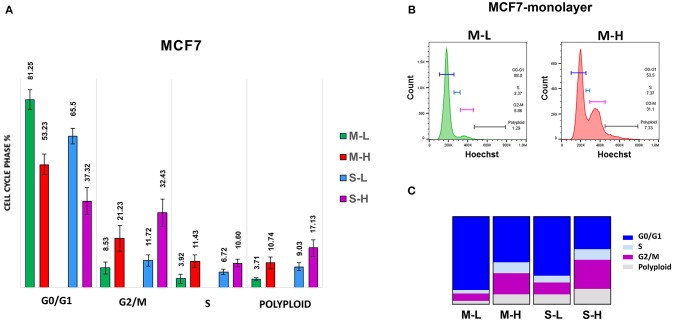
e-CSCs demonstrate increased cell cycle progression. Note that MCF7 cells with the highest flavin-content, have the highest rates of cell cycle progression and polyploidy. For example, M-H cells (from 2D-monolayers) and S-H cells (from 3D-spheroids) are hyper-proliferative and show the highest rates of cell cycle progression, as compared to the M-L and S-L sub-populations. Virtually identical results were also obtained when measuring the polyploidy of the various cell sub-populations; S-H had the highest numbers of polyploid cells. **(A)** Quantitation of cell cycle progression is shown as bar graphs, with the mean ± SD. **(B)** Representative traces from flow-cytometry for M-L and M-H sub-populations. **(C)** Condensed representation of **(A)**. See also Table 1 for comparison.

First, we assessed their capacity for cell proliferation, via cell cycle progression analysis. Representative cell cycle profiles for different cell sub-populations of MCF7 are shown in Figure 5. Interestingly, the M-H cell and S-H cell sub-populations were exceedingly hyper-proliferative, with a reduction of cells in the G0/G1-phase and dramatic increases in both the S-phase and the G2/M-phase. Also, the number of polyploid cells (DNA >2N) was increased considerably in both the M-H and S-H populations. Overall, S-H cells were the most hyper-proliferative, with >40% of the cells in S-phase and/or G2/M, and <40% of the cells in the G0/G1-phase of the cell cycle. S-H cells also had the largest number of polyploid cells, reaching ~12–17%, probably due to mitotic catastrophe.

In contrast, M-L cells had the highest number of cells in the G0/G1-phase of the cell cycle (~80%) and the lowest number of polyploid cells (~3–5%). Also, M-L cells showed the lowest number of cells in S-phase (~3–4%).

These hyper-proliferative results with MCF7 cells (Table 1; Figure 5) and MDA-MB-468 cells (See Table 2; Figure S1) are also consistent with a high-energy phenotype. Therefore, they were designated as “energetic” CSCs.

**Table 2:** MDA-MB-468 e-CSCs demonstrate increased cell cycle progression.

**CC-Phase (%)**	**2D-Monolayers (M)**		**3D-Spheroids (S)**	
	**M-L**	**M-H**	**S-L**	**S-H**
G0/G1	78.95	51.20	64.05	34.75
S-phase	2.96	12.18	9.03	18.10
G2/M	7.65	23.73	13.35	32.89
Polyploid	5.30	9.93	7.47	12.24

*Averages are shown from at least 3 independent experiments. M-L, monolayer-low; M-H, monolayer-high; S-L, spheroid-low; S-H, spheroid-high. See Figure S1 for specific SD*.

#### Stem Cell Characteristic(s)

**Figure 6:**
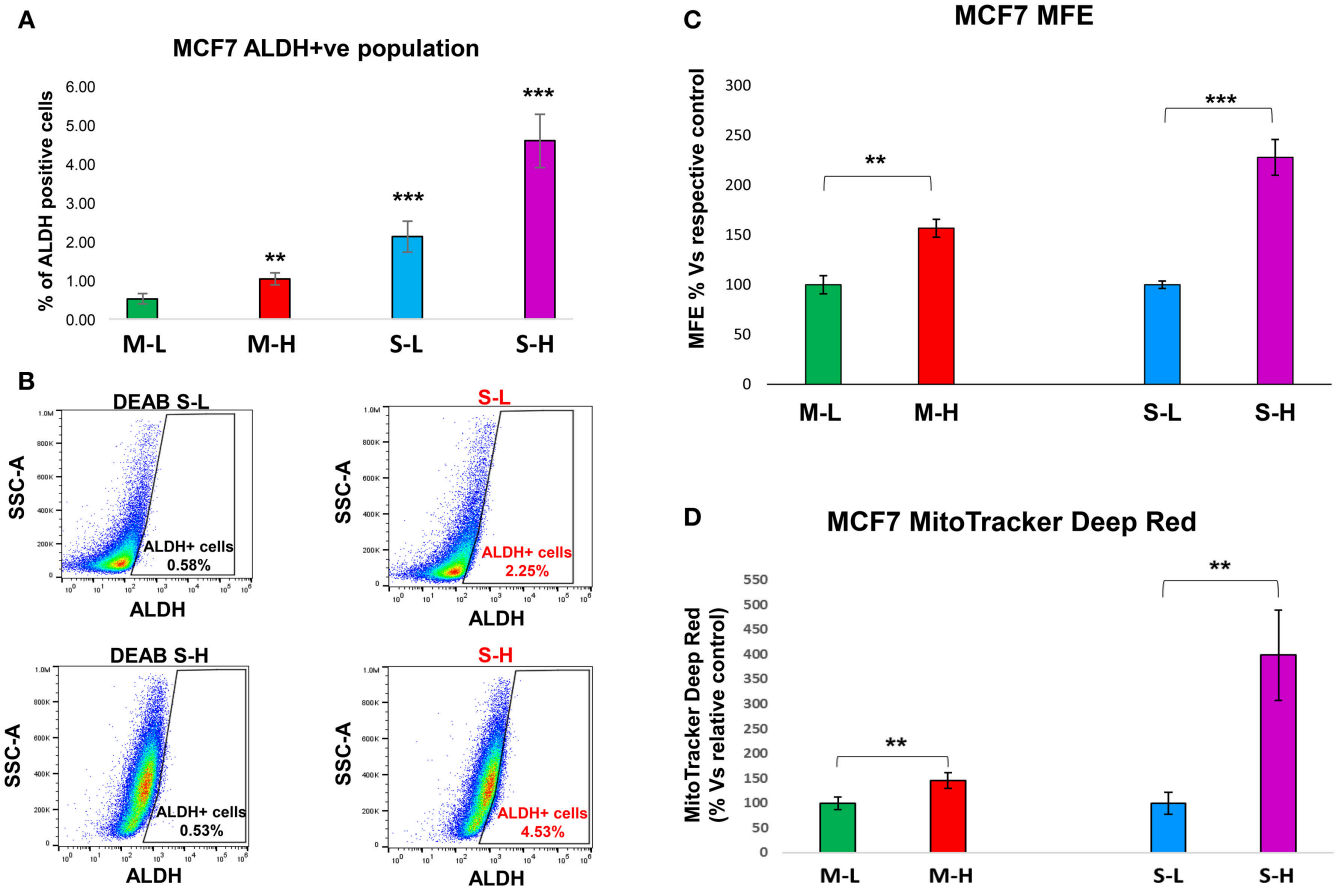
e-CSCs have increased “stem-like” features. **(A,B)** ALDH activity: Note that MCF7 cells with the highest flavin-content, have the highest ALDH activity. More specifically, M-H cells (from 2D-monolayers) and S-H cells (from 3D-spheroids) show the largest increases in ALDH activity, as seen by flow-cytometry analysis. Finally, the S-H sub-population had the highest amount of ALDH signal. Representative images are presented in **(B)**. **(C)** Anchorage-independent growth: The mammosphere assay allows for the quantitative measurement of anchorage-independent growth, which is a functional read-out for “stemness” activity. Note that high mammosphere formation in MCF7 cells directly correlates with high-flavin content. For example, M-H cells (from 2D-monolayers) and S-H cells (from 3D-spheroids) show the highest rates of mammosphere formation, as compared to the M-L and S-L sub-populations. **(D)** Mitochondrial mass (MitoTracker): Note that mitochondrial mass in MCF7 cells also correlates with high-flavin content. In particular, S-H cells (from 3D-spheroids) show that largest increases in mitochondrial mass, as seen by flow-cytometry with MitoTracker Deep Red vital staining. **p < 0.001 and ***p < 0.0001.

To carefully monitor the progressive enrichment of CSCs, we first used ALDH as a marker of “stemness” activity. Figures 6A,B clearly shows that relative to the cells with the least flavin (M-L), all the other cells showed the progressive enrichment of ALDH activity. Remarkably, M-H cells and S-H cells showed a 2-fold and a near 9-fold enrichment of ALDH-activity, respectively (Table 3). Their stem-like phenotype was further validated by using the mammosphere assay to measure anchorage-independent growth and by quantitatively measuring their mitochondrial mass, with a vital dye (namely, MitoTracker Deep Red).

**Table 3:** MCF7-derived e-CSCs have increased ALDH activity.

**2D-Monolayers (M)**		**3D-Spheroids (S)**	
**M-L**	**M-H**	**S-L**	**S-H**
0.52%	1.03% (1.98x)	2.13% (4.09x)	4.59% (8.83x)

*Averages are shown from 3 independent experiments. M-L, monolayer-low; M-H, monolayer-high; S-L, spheroid-low; S-H, spheroid-high. See Figure 6A for specific SD*.

Figure 6C illustrates that relative to control cells, the M-H and S-H cell sub-populations formed mammospheres with greater efficiency, ~1.6- and 2.3-fold, respectively. Figure 6D shows their mitochondrial status. Relative to M-L cells, M-H cells showed a clear ~1.45-fold increase in mitochondrial mass. Interestingly, relative to S-L cells, S-H cells demonstrated a remarkable ~4-fold increase in mitochondrial mass.

Therefore, e-CSCs derived from 3D-spheroids were the (i) most hyper-proliferative and showed the (ii) largest increases in stemness characteristics (ALDH activity and anchorage-independent growth), as well as the (iii) highest mitochondrial mass. These phenotypic changes are highly suggestive of metabolic re-programming, especially toward more oxidative mitochondrial metabolism.

#### Bioenergetics

To begin to characterize the bioenergetic phenotype of e-CSCs, they were subjected to metabolic flux analysis, using the Seahorse XFe96. We measured the mitochondrial oxygen consumption rate (OCR) and the extracellular acidification rate (ECAR).

**Figure 7:**
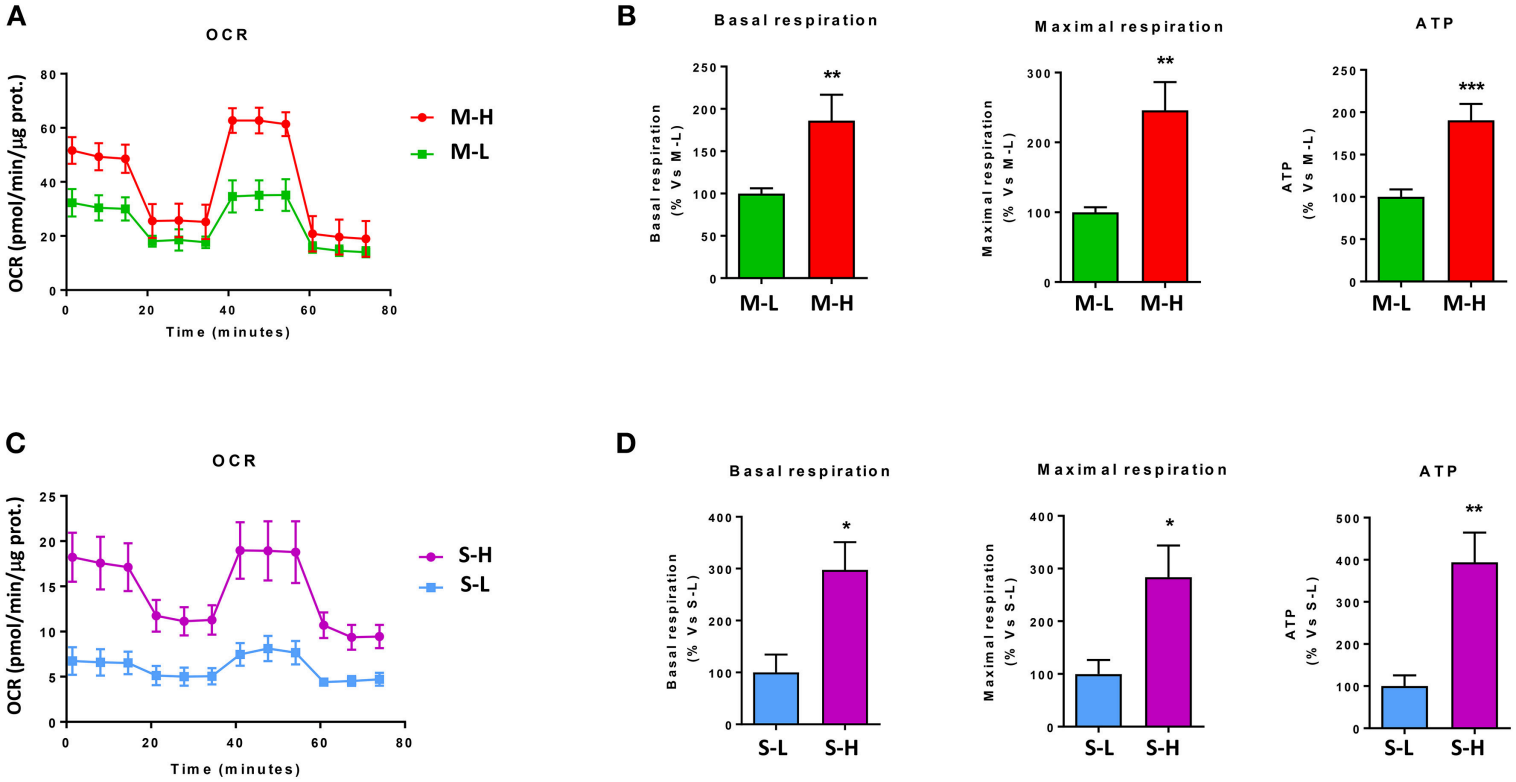
e-CSCs have increased oxidative mitochondrial metabolism. The oxygen consumption rate (OCR) was measured, using the Seahorse XFe96 metabolic-flux analyzer. Note that high OCR in MCF7 cells directly correlates with high-flavin content. For example, M-H cells (from 2D-monolayers) and S-H cells (from 3D-spheroids) have the highest levels of OCR, as compared to the M-L and S-L sub-populations. **(A,B)** OCR for M-L vs. M-H sub-populations; **(C,D)** OCR for S-L vs. S-H sub-populations. *p < 0.01, **p < 0.001 and ***p < 0.0001.

**Figure 8:**
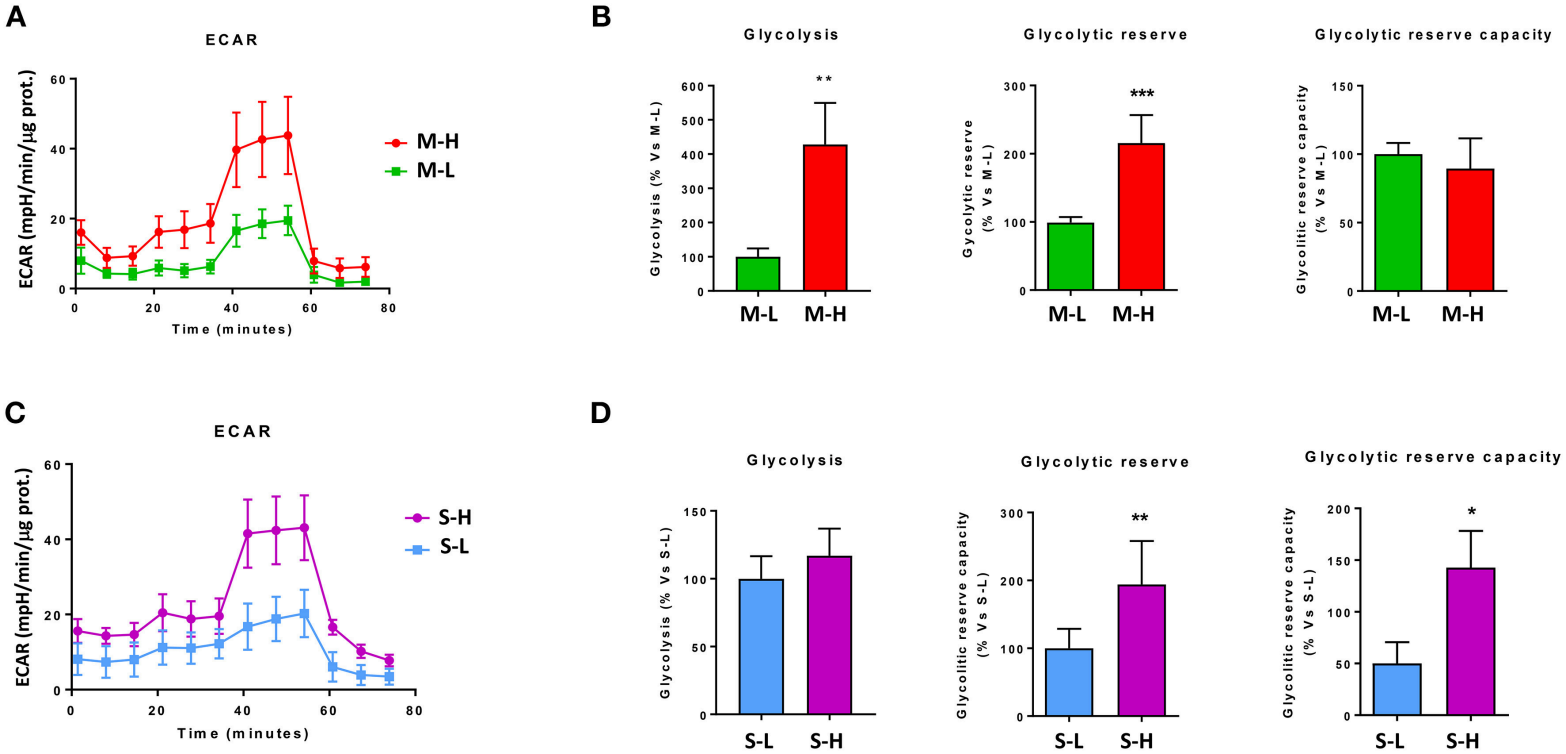
e-CSCs have elevated levels of aerobic glycolysis. The extracellular acidification rate (ECAR) was measured, using the Seahorse XFe96 metabolic-flux analyzer. Note that high ECAR in MCF7 cells directly correlates with high-flavin content. For example, M-H cells (from 2D-monolayers) and S-H cells (from 3D-spheroids) have the highest levels of ECAR, as compared to the M-L and S-L sub-populations. **(A,B)** ECAR for M-L vs. M-H sub-populations; **(C,D)** ECAR for S-L vs. S-H sub-populations. *p < 0.01, **p < 0.001 and ***p < 0.0001.

Figure 7A shows that M-H cells are highly oxidative, with a near 2-fold increase in OCR, mitochondrial respiration and ATP-production. However, the largest changes were in their glycolytic phenotype, with an ~4-fold increase in glycolytic activity (Figure 8A). As such, M-H cells are highly glycolytic, but with enhanced mitochondrial metabolism.

In contrast, S-H cells demonstrated the highest increases in OCR, with a near 3-fold increase in basal respiration and a 4-fold increase in ATP production (Figure 7B). However, their basal glycolytic rate remained unchanged, suggestive of a greater dependence on mitochondrial OXPHOS metabolism (Figure 8B). As a consequence, S-H cells may be more sensitive to mitochondrial OXPHOS inhibitors, highlighting a possible weak point in e-CSCs derived from 3D-spheroids.

### Targeting e-CSCs With OXPHOS Inhibitors and CDK4/6 Inhibitors

#### DPI Treatment

**Figure 9:**
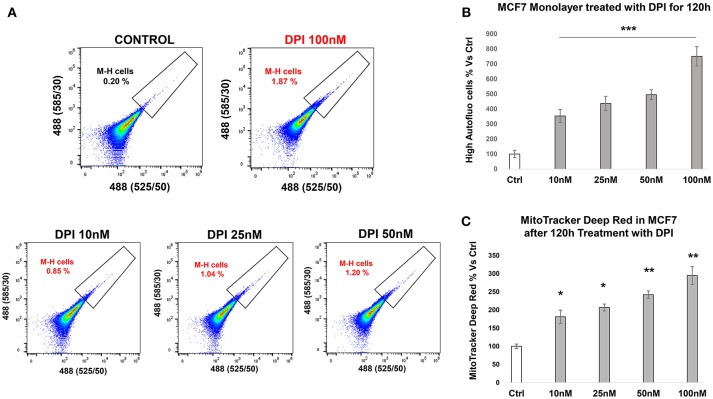
e-CSCs (2D) propagate in response to DPI, an OXPHOS inhibitor. **(A)** Representative tracings from flow-cytometry, monitoring the increase in the M-H cell sub-population. **(B)** The bar graph shows that the percentage of M-H cells are increased after treatment with DPI, a mitochondrial OXPHOS inhibitor, over a 5-days period, in a concentration dependent manner. **(C)** The bar graph shows that the mitochondrial mass (MitoTracker) is increased after treatment with DPI, over the same time-frame. *p < 0.01, **p < 0.001 and ***p < 0.0001.

**Figure 10:**
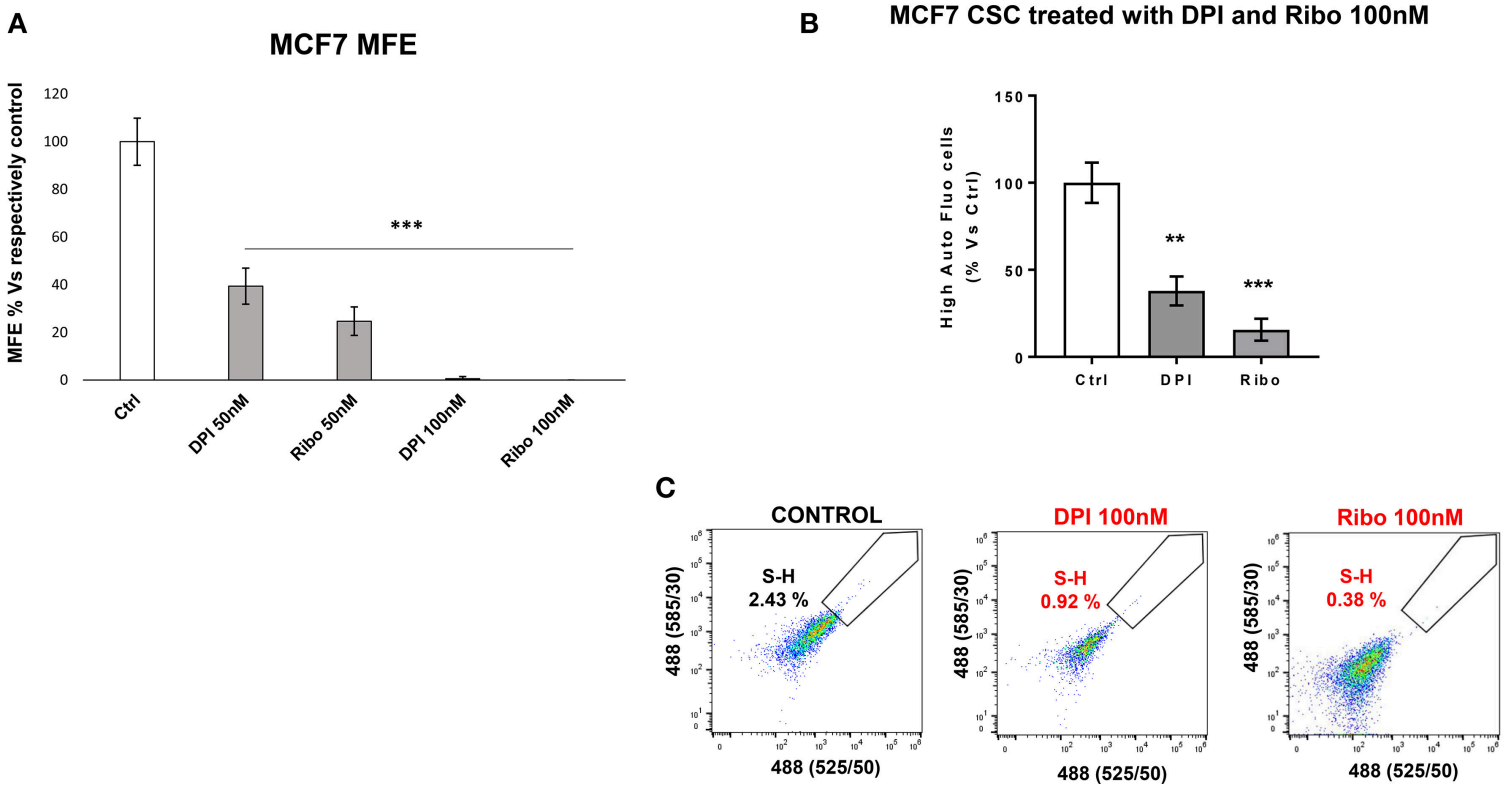
e-CSCs (3D) are susceptible to targeting with DPI or Ribociclib. **(A)** The bar graph shows that mammosphere formation in MCF7 cells is inhibited in response to DPI or Ribociclib treatment, in a concentration dependent-manner (0, 50, and 100 nM). **(B)** Note that the percentage of S-H cells are reduced after treatment with either DPI or Ribociclib. **(C)** Representative flow-cytometry experiments are also shown, as supporting evidence. Therefore, DPI has opposite effects on M-H and S-H cells. As such, DPI selectively targets the S-H sub-population of e-CSCs. **p < 0.001 and ***p < 0.0001.

DPI (Diphenyleneiodonium chloride) is an OXPHOS inhibitor that specifically targets flavin-containing enzymes, especially those associated with FMN/FAD and mitochondrial complex I and II (8–10). We have previously demonstrated that DPI inhibits 3D-spheroid formation in MCF7 cells (28). We previously documented that DPI selectively inhibits mitochondrial function without any toxic side effects; it did not induce changes in cell viability or apoptosis, but shifted the cells toward a more glycolytic phenotype (28). Interestingly, Figure 9 reveals that M-H cells treated with DPI show significant increases in their propagation (Figures 9A,B) and mitochondrial mass (Figure 9C). This is consistent with their high basal glycolytic rate. In contrast, S-H cells were sensitive to DPI; it inhibited their anchorage-independent growth and propagation in a dose-dependent fashion (Figure 10).

Therefore, e-CSCs are metabolically-wired differently, depending on if they are proliferating in a 2D-monolayer or a 3D-spheroid micro-environment. Most importantly, e-CSCs derived from 3D-spheroids are highly oxidative and can be effectively targeted with an OXPHOS inhibitor.

#### Ribociclib Treatment

Complementary experiments were also carried out with Ribociclib, a clinically-approved CDK4/6 inhibitor. Ribociclib is normally used to treat female breast cancer patients, in combination with letrozole (an aromatase inhibitor) (29–34). Ribociclib was first developed by Astex Pharmaceuticals (Cambridge, UK) and Novartis (35). In 2017, Ribociclib was approved by the FDA and the European Medicines Agency, for the treatment of HR-positive, HER2-negative advanced or metastatic breast cancers (36). The drug's most common side-effects are: neutropenia, anemia and GI-distress.

Figure 10 illustrates that treatment with Ribociclib effectively inhibits the propagation of S-H cells. Therefore, anchorage-independent proliferation by S-H cells is critically-dependent on CDK4/6 function, as well as mitochondrial metabolism.

### Proteomics Analysis of e-CSCs, Derived From MCF7 3D-Spheroids

To begin to understand the mechanistic basis for the biogenesis of e-CSCs, we next subjected them to label-free unbiased proteomics analysis. As a consequence, we identified 225 proteins that were significantly up-regulated by >1.5-fold. Conversely, 187 proteins were significantly down-regulated.

For simplicity, we focused on the specific protein products that were up-regulated and these are highlighted in Table S1. Interestingly, 48 of these proteins (representing ~20% of the total number) were specifically related to mitochondrial energy production and/or mitochondrial biogenesis (See Table S2). This is consistent with our functional observations that e-CSCs demonstrate a near 4-fold increase in both (i) mitochondrial mass and (ii) mitochondrial ATP production.

Table S3, shows further bioinformatics analysis, assembling the proteins into distinct functional groups. These functional classes include senescence, the anti-oxidant response, “stemness,” cytoskeletal proteins (suggestive of an EMT), glutamine metabolism, NADH/NADPH synthesis, flavin-containing enzymes, autophagy/lysosomes, peroxisomes, and various cellular markers (epithelial, cell surface, S100 family proteins, RABs, annexins, PARP, calcium signaling).

Interestingly, CDKN1A (p21 WAF), which is a CDK-inhibitor and senescence marker, is highly up-regulated by 17.22-fold in e-CSCs. This finding is consistent with the idea that CSCs originate from senescent cells (37–40).

However, e-CSCs are hyper-proliferative, so they must have successfully escaped from senescence. We speculate that this may have occurred through the over-expression of anti-oxidant enzymes or the over-production of NADH/NADPH. Loss of glutaredoxin expression is known to be sufficient to induce a senescence phenotype in cells, in a p21-dependent manner. Therefore, the observed over-expression of glutaredoxin (by 10.79-fold) may be sufficient to actually overcome senescence, allowing the creation of e-CSCs.

Importantly, glutaredoxin expression is known to drive mitochondrial biogenesis by directly regulating the activation state of two key mitochondrial proteins, namely HSP60 and DJ-1 (Park7) (37). HSP60 is a mitochondrial chaperone, which facilitates the proper folding of newly synthesized or imported mitochondrial proteins, while DJ-1 functionally maintains the activity of mitochondrial complex I and SOD2. As a consequence, glutaredoxin expression specifically maintains the integrity of mitochondria and elevates ATP synthesis (37). Glutaredoxin's ability to regulate mitochondrial energy production is also linked to cell cycle progression. As such, glutaredoxin allows cells to successfully pass through the G1/S transition, in a CDK4-dependent manner, thereby avoiding the cell-cycle arrest associated with senescence (37).

The 10.24-fold up-regulation of ALDH3A1 also provides significant anti-oxidant power, as ALDH isoforms are known to functionally increase the cellular levels of NADH/NADPH (41–45). Also, the main isoform up-regulated in e-CSCs, namely ALDH3A1, is known to be associated with tumorigenesis, metastasis and drug-resistance (41–45).

**Figure 11:**
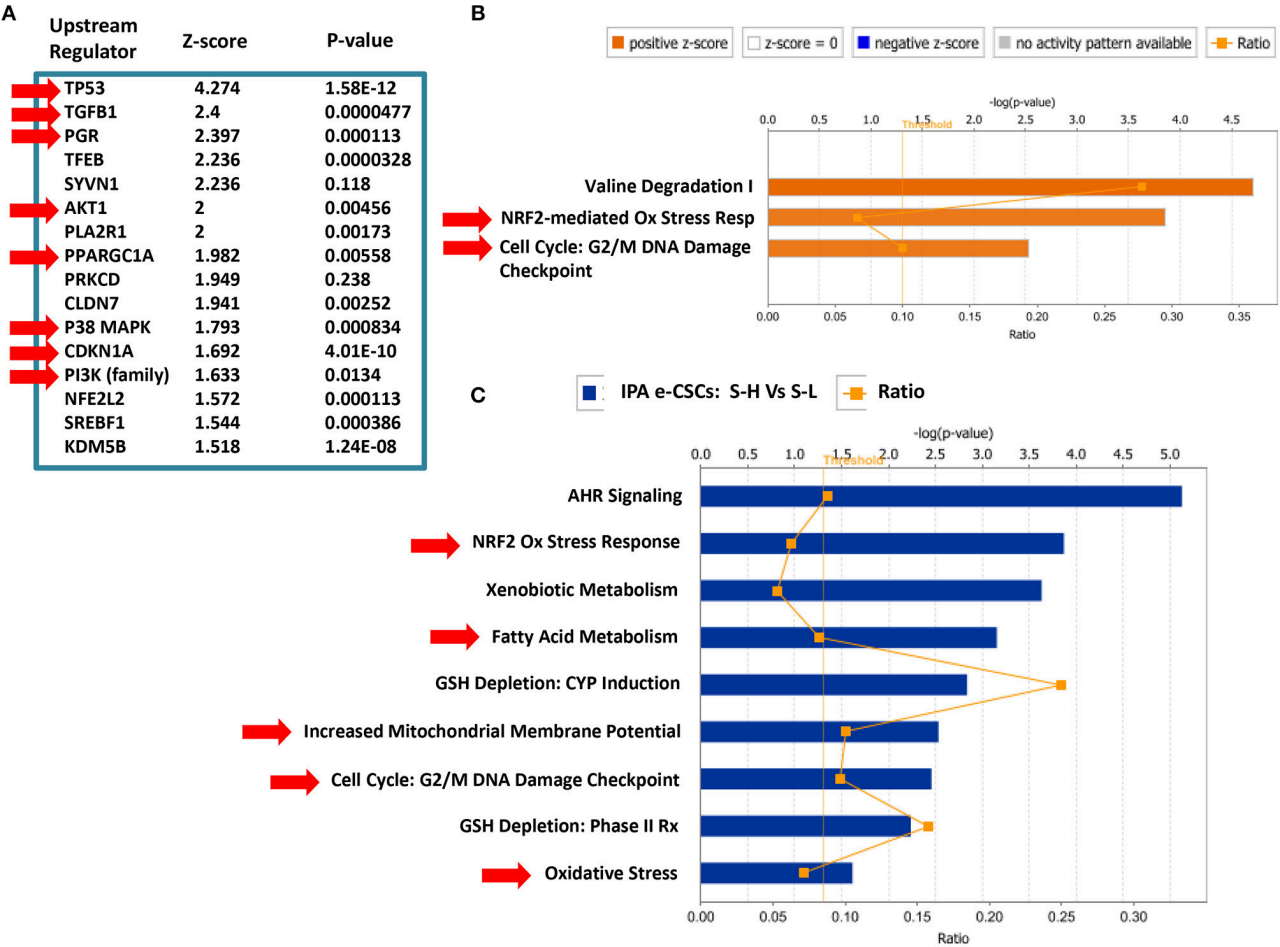
Ingenuity Pathway Analysis (IPA) of the Proteomics Data from MCF7 e-CSCs, Derived from 3D-Spheroids. IPA of our proteomics data sets (S-H vs. S-L) confirms the up-regulation the anti-oxidant response (NRF2) and cell cycle progression in e-CSCs, as well as the changes in mitochondrial function and fatty acid metabolism (see RED arrowheads). Note the activation of PPARGC-1-Alpha, also known as PGC-1-Alpha, the major mitochondrial transcription factor. **(A)** Upstream regulator analysis; **(B)** canonical pathways analysis; **(C)** toxicology list analysis. In this analysis, the upstream regulator CD24 was inhibited/down-regulated in e-CSCs, with a Z-score of -2.22 (*p* < 0.05) [data not shown]. In addition, in panel **(A)**, note that p53, TGFB1, PGR (progesterone receptor), AKT1/PI3K, CDKN1A (p21 WAF), and p38 MAPK signaling were all activated/up-regulated in e-CSCs.

Interestingly, IPA of our proteomics data sets indeed confirms the up-regulation the anti-oxidant response and cell cycle progression in e-CSCs, as well as the changes in mitochondrial function (Figure 11). Note the activation of PPARGC-1-Alpha, also known as PGC-1-Alpha, the major mitochondrial transcription factor.

### e-CSC Proteins Are Transcriptionally Up-Regulated in Human Breast Cancer Patients

To determine if e-CSCs proteins were also transcriptionally up-regulated in human breast cancer cells *in vivo*, we next interrogated pre-existing mRNA profiling data, obtained from the laser-capture analysis of *N* = 28 human breast cancer patient tumor samples. In this data set, breast cancer cells were physically separated from adjacent tumor stromal cells, using laser-captured mediated micro-dissection.

The results of this intersection are presented in Table S4. Briefly, our results indicate that out of the 225 proteins that were up-regulated in e-CSCs, nearly one-third of these gene products were transcriptionally up-regulated in human breast cancer cells (70/225 = 31.11%). In addition, many of these gene products that were shared, included 20 mitochondrial related genes (20/70 = 28.57%).

These results provide genetic evidence to support the potential clinical relevance of e-CSCs in the study of human breast cancers.

### A Short Anti-oxidant Response Signature in e-CSCs Predicts Poor Clinical Outcome in Breast Cancer Patients

We next determined if a subset of e-CSCs proteins have prognostic value in terms of predicting clinical outcome in human breast cancer patients. For this purpose, we used a well-defined set of high-risk ER(+) patients (luminal A) that received hormone-therapy (mostly-tamoxifen), with local Lymph-Node (LN) metastasis at diagnosis, as well as >150 months (12.5 years) of follow-up data. In all of these cancer patients, their breast tumor tissues also underwent genomic transcriptional profiling.

Using Kaplan-Meier (K-M) analysis, we specifically determined if these e-CSCs proteins (listed in Table S4) had prognostic value by determining their effects on the Hazard-Ratio (HR), by employing the Log-Rank test to determine statistical significance. Based on this analysis, we defined a four-gene signature consisting of members of the anti-oxidant response and NAD(P)H metabolism, namely NQO1, ALDH5A1, TXNR, and RRM2 (Table S5). Importantly, the other 66 gene products tested did not show this prognostic ability.

First, these four gene products were tested individually and all showed a >2-fold increase in the HR. In addition, when combined into a short signature, this resulted in a HR of nearly 4, with a *p*-value of 4.1e-05. As a consequence, this anti-oxidant signature could be used to predict tumor recurrence (RFS) in patients receiving hormonal therapy (Table S5).

Similarly, we also observed that the transcriptional elevation of 3 out of 4 of these gene products (ALDH5A1, TXNR and RRM2) was effectively able to predict distant metastasis (DMFS) (Table S6), with HRs of 2.86–3.64, and *p*-values of 0.003–0.00035.

Therefore, the anti-oxidant response in e-CSCs has allowed us to successfully identify gene products with predictive value, for anticipating the onset of (i) recurrence and (ii) metastasis in breast cancer patients that ultimately underwent treatment failure, in response to hormonal therapy.

K-M curves for larger groups of breast cancer patients are also shown in Figure S4, which all showed significant prognostic value. These included patients that were ER(+) (*N* = 2,780) and ER(–) (*N* = 791), as well as all breast cancer sub-types, taken together (*N* = 3,571). Therefore, this signature should have broad applicability in breast cancer and possibly other cancer types.

One explanation for the prognostic value of this compact gene signature is that an adaptive anti-oxidant response drives resistance to both chemotherapy and radiotherapy in cancer patients (46, 47). In addition, TXNR and RRM2 both are key enzymes that provide the required precursors for nucleotide biosynthesis and, hence, cell cycle progression.

We speculate that this anti-oxidant response signature would also be useful for identifying breast cancer patients that could benefit from treatment with (i) mitochondrial inhibitors or (ii) CDK inhibitors, especially in the context of preventing tumor recurrence. Thus, in the future, it will be interesting to assess ROS production in e-CSCs, under both 2D and 3D microenvironmental conditions, to validate that ROS production is driving this anti-oxidant response signature and contributes to their overall energetic phenotype.

## Discussion

Here, we successfully purified a hyper-proliferative cell sub-population of breast CSCs, by using an endogenous marker of energy-metabolism, namely, flavin-derived auto-fluorescence. To better describe this new cell phenotype, we coined the term “energetic” CSCs (e-CSCs).

**Figure 12:**
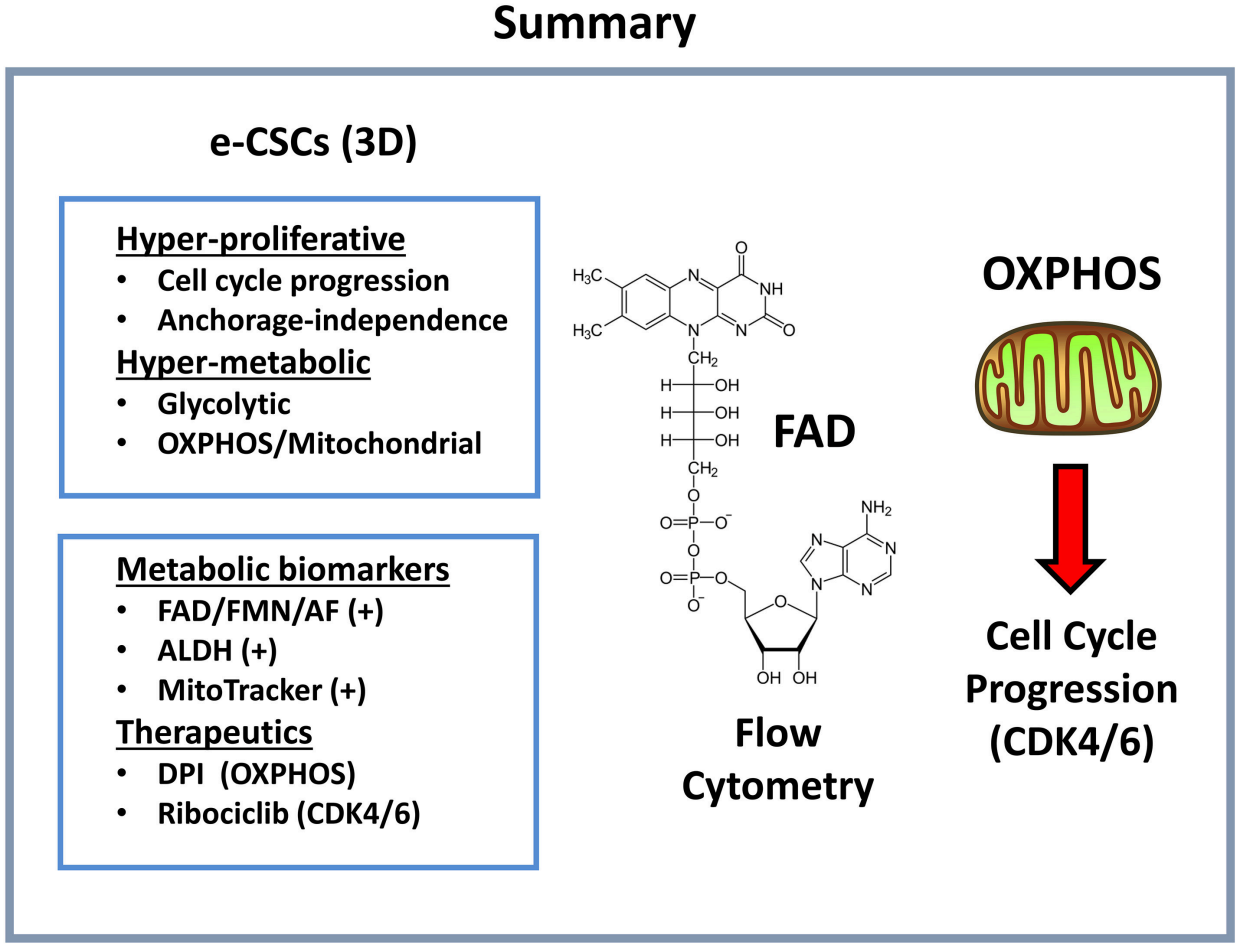
Summary: Functional characteristics of e-CSCs. Here, we report a new cancer cell phenotype, directly linking cell energy metabolism with proliferation. e-CSCs were highly energetic, with elevations both in glycolysis and oxidative mitochondrial metabolism. In addition, e-CSCs showed increased cell cycle progression, with an enhanced capacity for anchorage-independent growth. More specifically, e-CSCs were enriched in the following three functional biomarkers: AF(+), ALDH(+), and MitoTracker(+). Interestingly, the propagation of e-CSCs (3D) was halted by treatment with (i) OXPHOS inhibitors (DPI) or (ii) inhibitors of cell cycle progression (Ribociclib). DPI functionally induces a Vitamin B2 deficiency by targeting flavin-containing enzymes; Ribociclib is a CDK4/6 inhibitor. AF, auto-fluorescent.

**Figure 13:**
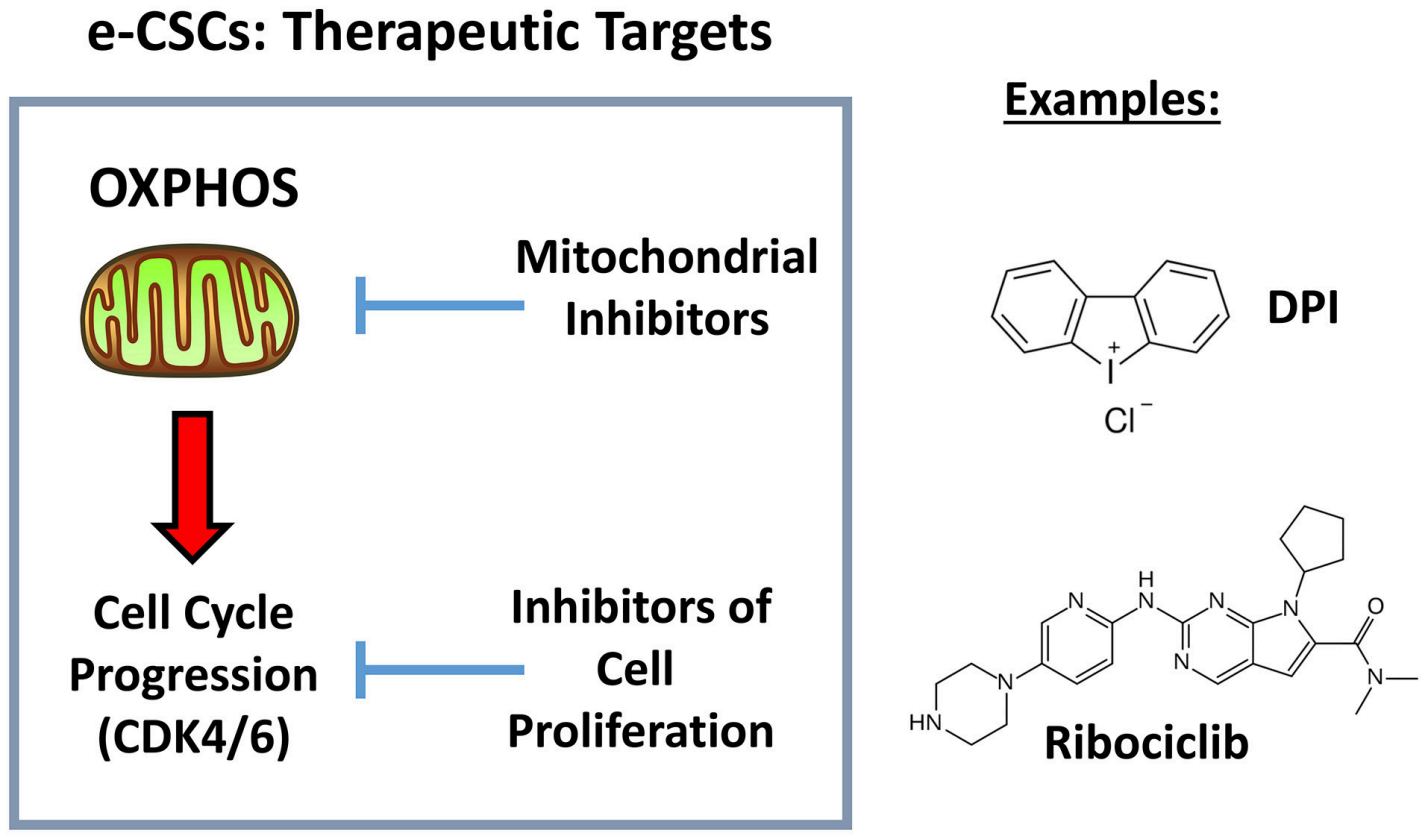
Therapeutic targets in e-CSCs (3D): OXPHOS and CDK4/6. Here, we identified a new cancer cell phenotype, linking cell proliferation with mitochondrial metabolism. Importantly, we also show that both mitochondrial OXPHOS inhibitors (DPI) and inhibitors of cell proliferation (Ribociclib) can be used to effectively target e-CSCs.

More specifically, in addition to a hyper-proliferative phenotype, e-CSCs showed progressive increases in stemness markers (ALDH activity and mammosphere-forming activity), a highly elevated mitochondrial mass, as well as increased glycolytic and mitochondrial activity. This new CSC phenotype is summarized schematically in Figure 12. Moreover, we directly demonstrated that the anchorage-independent propagation of e-CSCs, derived from 3D-spheroids, could be specifically targeted with an OXPHOS inhibitor (DPI) or a CDK4/6 inhibitor (Ribociclib) (Figure 13). Therefore, this 3D sub-type of e-CSCs is strictly dependent on mitochondria, for cell propagation.

Mechanistically, we propose that there are at least 2 different classes of e-CSCs, that are metabolically distinct and that this is dependent on whether they are grown in a 2D-monolayer or a 3D-spheroid micro-environment. We observed that a metabolic-switch is occurring, apparently during the transition from anchorage-dependent to anchorage-independent growth. This represents a metabolic shift from a glycolytic to a more oxidative mitochondrial phenotype.

More specifically, in 2D-monolayer cultures, 100 nM DPI increased the number of M-H cells by ~7.5-fold over a 5-days period. In contrast, DPI, at exactly the same concentration, almost completely inhibited 3D-mammosphere formation, resulting in a population of anchorage-independent single live cells that were ~60% depleted of S-H cells. Therefore, the same mitochondrial OXPHOS inhibitor (DPI) had completely opposite effects, depending on the 2D vs. 3D micro-environment of the e-CSCs.

These results experimentally imply that M-H cell propagation in 2D-monolayers is driven by glycolysis, while the propagation of S-H cells in 3D-spheroids is driven by mitochondrial OXPHOS. Importantly, this would suggest that a critical metabolic-switch is occurring, between the M-H and S-H CSC phenotypes, specifically altering their metabolic requirements.

**Figure 14:**
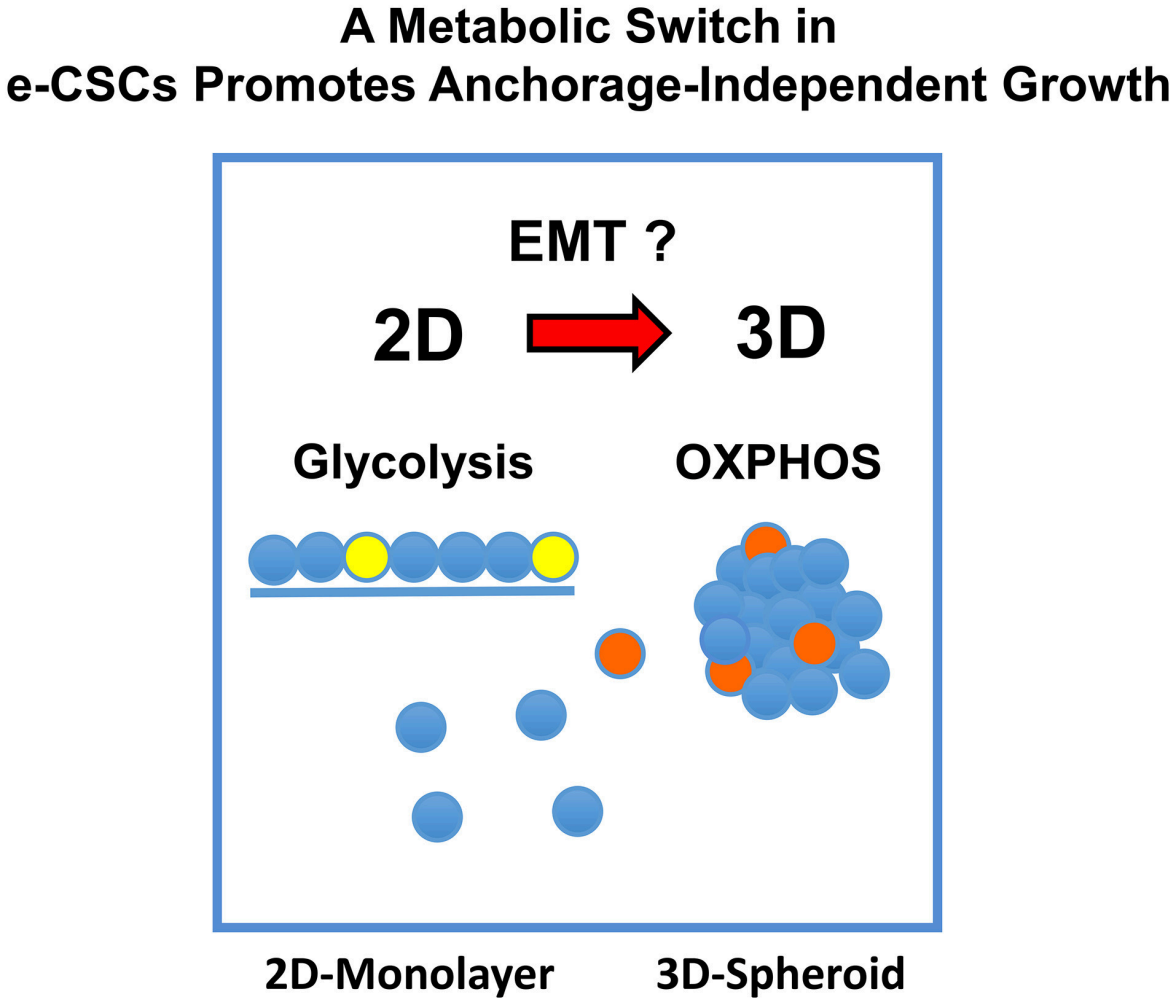
A metabolic-switch promotes anchorage-independent growth in e-CSCs. In 2D-monolayer cultures, e-CSCs are particularly dependent on glycolysis to maintain a high rate of cell proliferation. Experimentally, their rate of propagation is increased by an OXPHOS inhibitor (DPI). In striking contrast, in 3D-spheroid cultures, e-CSCs strictly require mitochondrial OXPHOS to undergo anchorage-independent growth. This would explain our results with metabolic flux analysis, using the M-H and S-H cell sub-populations, and their differential sensitivity to DPI-treatment. Therefore, the development and implementation of anti-mitochondrial therapies may be a viable strategy for preventing the onset of tumor metastasis, which involves an anchorage-independent growth phase as a required step.

This so called 2D-to-3D transition or “epithelial-mesenchymal-transition (EMT)” is thought to be a more mesenchymal phenotype. In support of this notion, ALDH activity was progressively increased and was at its highest levels in e-CSCs derived from the 3D-spheroids, nearly 9-fold increased, directly supporting our assertions (Figure 14). Importantly, ALDH activity is an established functional biomarker of the EMT and “boosts” the production of energy-rich NAD(P)H (17, 48–50).

The identification of this unique, energy-driven, cancer cell sub-population will undoubtedly provide new opportunities for (i) bio-banking and (ii) new drug screening, as well as (iii) the identification of novel metabolic targets, for the prevention of tumor recurrence and inhibiting the spread of metastatic disease.

It has long remained a mystery, what is the exact nature of the cancer cell of origin (51–55). While this still remains a mystery, we would like to speculate that our current findings, which are based on functional and operational definitions, will help to provide an additional practical framework for studying CSCs, more from a proliferative and energetic perspective.

## Author Contributions

ML, FS, and MF conceived and initiated this project. All experiments described in this paper were performed by MF, who then generated the final figures and tables for the paper. ML and FS wrote the first draft of the manuscript, which was then further edited by MF. ML generated the schematic summary diagrams.

### Conflict of Interest Statement

ML and FS hold a minority interest in Lunella Biotech, Inc. The remaining author declares that the research was conducted in the absence of any commercial or financial relationships that could be construed as a potential conflict of interest.
