# Supplementary material for: “Energetic” Cancer Stem Cells (e-CSCs): A New Hyper-Metabolic and Proliferative Tumor Cell Phenotype, Driven by Mitochondrial Energy
Source: Front Oncol. 2019 Feb 5;8:677. doi: 10.3389/fonc.2018.00677 (PMC6370664; doi:10.3389/fonc.2018.00677)
Supplement: Supplementary file 1 [file Data_Sheet_1.pdf]

Supplemental Material (Tables S1-S6)

Table S1: Proteomic analysis of e-CSCs, derived from MCF7 3D-Spheroids.

Symbol	Gene Description	Fold-Change (Up-regulation)
BCAS1	Breast carcinoma-amplified sequence 1	119.37
CDKN1A	Cyclin-dependent kinase inhibitor 1 (p21-WAF/CDK-inhibitor)	17.22
GLRX	Glutaredoxin-1	10.79
ALDH3A1	Aldehyde dehydrogenase, dimeric NADP-preferring	10.24
CEACAM6	Carcinoembryonic antigen-related cell adhesion molecule 6	9.66
CYP1A1	Cytochrome P450 1A1	6.60
ELMOD2	ELMO domain-containing protein 2	4.73
MAOA	Amine oxidase [flavin-containing] A	4.73
KRT10	Keratin, type I cytoskeletal 10	4.59
IGFBP2	Insulin-like growth factor-binding protein 2	4.20
QPRT	Nicotinate-nucleotide pyrophosphorylase [carboxylating]	3.72
MVP	Major vault protein	3.61
CEACAM5	Carcinoembryonic antigen-related cell adhesion molecule 5	3.38
CLU	Clusterin	3.13
QSOX1	Sulfhydryl oxidase 1	2.93
CIB1	Calcium and integrin-binding protein 1	2.90
VGF	Neurosecretory protein VGF	2.90
ANXA1	Annexin A1	2.87
AKR1C3	Aldo-keto reductase family 1 member C3	2.79
LAMA5	Laminin subunit alpha-5	2.72
CDC42BPG	Serine/threonine-protein kinase MRCK gamma	2.69
RAB27B	Ras-related protein Rab-27B	2.69
CHMP6	Charged multivesicular body protein 6	2.62
TUBA4A	Tubulin alpha-4A chain	2.60
PARP4	Poly [ADP-ribose] polymerase 4	2.55

RAB27A	Ras-related protein Rab-27A	2.54
EVPL	Envoplakin	2.48
KLK11	Kallikrein-11	2.46
MAOB	Amine oxidase [flavin-containing] B	2.45
DPP7	Dipeptidyl peptidase 2	2.43
AKR1C2	Aldo-keto reductase family 1 member C2	2.41
SFXN3	Sideroflexin-3	2.40
MIC13	MICOS complex subunit MIC13, mitochondrial	2.36
GM2A	Ganglioside GM2 activator	2.36
SCRN2	Secernin-2	2.34
SULT1A1	Sulfotransferase 1A1	2.34
RRM2	Ribonucleoside-diphosphate reductase subunit M2	2.34
SERPINA3	Alpha-1-antichymotrypsin	2.33
SLC6A14	Sodium - and chloride-dependent neutral and basic amino acid transporter B(0+)	2.30
AGA	N(4)-(beta-N-acetylglucosaminyl)-L-asparaginase	2.30
SYTL2	Synaptotagmin-like protein 2	2.30
MPV17	Protein Mpv17	2.28
KIAA0319L	Dyslexia-associated protein KIAA0319-like protein	2.25
B3GAT3	Galactosylgalactosylxylosylprotein 3-beta-glucuronosyltransferase 3	2.22
PON2	Serum paraoxonase/arylesterase 2	2.22
OXSM	3-oxoacyl-[acyl-carrier-protein] synthase, mitochondrial	2.22
TOM1L2	TOM1-like protein 2	2.22
STOM	Erythrocyte band 7 integral membrane protein	2.18
MROH1	Maestro heat-like repeat-containing protein family member 1	2.17
PI4K2A	Phosphatidylinositol 4-kinase type 2-alpha	2.17
FECH	Ferrochelatase, mitochondrial	2.16
MCU	Calcium uniporter protein, mitochondrial	2.13
S100P	Protein S100-P	2.11
RDH13	Retinol dehydrogenase 13	2.08
PPL	Periplakin	2.08
TSPAN31	Tetraspanin-31	2.03
TIMP1	Metalloproteinase inhibitor 1	2.02

GCLC	Glutamate--cysteine ligase catalytic subunit	2.01
NEBL	Nebulette	2.01
MUC5B	Mucin-5B	1.98
CTSH	Cathepsin H	1.98
GNS	N-acetylglucosamine-6-sulfatase	1.97
S100A10	Protein S100-A10	1.96
INPP4B	Type II inositol 3,4-bisphosphate 4-phosphatase	1.96
PHYKPL	5-phosphohydroxy-L-lysine phospho-lyase	1.95
ASAH1	Acid ceramidase	1.94
DHRS1	Dehydrogenase/reductase SDR family member 1	1.93
PEX14	Peroxisomal membrane protein PEX14	1.91
PTGR1	Prostaglandin reductase	1.91
NQO2	Ribosyldihydronicotinamide dehydrogenase [quinone]	1.90
STARD3NL	STARD3 N-terminal-like protein	1.88
MGST1	Microsomal glutathione S-transferase	1.88
CMC1	COX assembly mitochondrial protein homolog	1.87
DGAT1	Diacylglycerol O-acyltransferase 1	1.87
RAB24	Ras-related protein Rab-24	1.87
GDPD3	Lysophospholipase D GDPD3	1.86
DCLK1	Serine/threonine-protein kinase DCLK1	1.85
PSAP	Prosaposin	1.85
MGST3	Microsomal glutathione S-transferase 3	1.84
ANO10	Anoctamin-10	1.84
CASK	Peripheral plasma membrane protein CASK	1.84
LGALS3BP	Galectin-3-binding protein	1.83
GAA	Lysosomal alpha-glucosidase	1.83
ISCU	Iron-sulfur cluster assembly enzyme ISCU, mitochondrial	1.83
GALNS	N-acetylgalactosamine-6-sulfatase	1.82
DECR2	Peroxisomal 2,4-dienoyl-CoA reductase	1.81
ABAT	4-aminobutyrate aminotransferase, mitochondrial	1.81
PALM3	Paralemmin-3	1.81
ABCB6	ATP-binding cassette sub-family B member 6, mitochondrial	1.80

GFER	FAD-linked sulfhydryl oxidase ALR	1.80
CD59	CD59 glycoprotein	1.80
SLC39A11	Zinc transporter ZIP11	1.80
CAPN2	Calpain-2 catalytic subunit	1.79
FAM174B	Membrane protein FAM174B	1.79
TMEM160	Transmembrane protein 160	1.79
ACADSB	Short/branched chain specific acyl-CoA dehydrogenase, mitochondrial	1.79
FAM8A1	Protein FAM8A1	1.79
CAPS	Calcyphosin	1.79
ARMC10	Armadillo repeat-containing protein 10	1.78
TMTC3	Transmembrane and TPR repeat-containing protein 3	1.78
SCFD2	Sec1 family domain-containing protein 2	1.78
HDHD3	Haloacid dehalogenase-like hydrolase domain-containing protein 3	1.78
RETSAT	All-trans-retinol 13,14-reductase	1.77
COQ9	Ubiquinone biosynthesis protein COQ9, mitochondrial	1.77
SPATA20	Spermatogenesis-associated protein 20	1.77
EML2	Echinoderm microtubule-associated protein-like 2	1.77
ALDH5A1	Succinate-semialdehyde dehydrogenase, mitochondrial	1.76
GRN	Granulins	1.76
CPT2	Carnitine O-palmitoyltransferase 2, mitochondrial	1.76
PEX11B	Peroxisomal membrane protein PEX11B	1.76
HMGCL	Hydroxymethylglutaryl-CoA lyase, mitochondrial	1.75
GSTK1	Glutathione S-transferase kappa 1	1.75
DHRS7B	Dehydrogenase/reductase SDR family member 7B	1.75
FDXR	NADPH:adrenodoxin oxidoreductase, mitochondrial	1.75
EPS8L1	Epidermal growth factor receptor kinase substrate 8-like protein 1	1.74
SLC22A18	Solute carrier family 22 member 18	1.74
CYCS	Cytochrome c	1.74
MAPRE3	Microtubule-associated protein RP/EB family member 3	1.74
SQOR	Sulfide:quinone oxidoreductase, mitochondrial	1.73
PDIA5	Protein disulfide-isomerase A5	1.73
HIGD1C	HIG1 domain family member 1C	1.72

EML3	Echinoderm microtubule-associated protein-like 3	1.72
PCLAF	PCNA-associated factor	1.72
ATP6V0A1	V-type proton ATPase 116 kDa subunit a isoform 1	1.71
TAOK3	Serine/threonine-protein kinase TAO3	1.71
ITGAV	Integrin alpha-V	1.71
CAMK2D	Calcium/calmodulin-dependent protein kinase type II subunit delta	1.70
SLC9A1	Sodium/hydrogen exchanger 1	1.69
CALML5	Calmodulin-like protein 5	1.69
HMOX1	Heme oxygenase 1	1.69
RNASET2	Ribonuclease T2	1.69
SELENBP1	Methanethiol oxidase	1.68
ACAA1	3-ketoacyl-CoA thiolase, peroxisomal	1.68
FKBP11	Peptidyl-prolyl cis-trans isomerase FKBP11	1.68
RRM2B	Ribonucleoside-diphosphate reductase subunit M2 B	1.68
MLYCD	Malonyl-CoA decarboxylase, mitochondrial	1.67
ENDOG	Endonuclease G, mitochondrial	1.67
HPDL	4-hydroxyphenylpyruvate dioxygenase-like protein	1.67
CYB5R1	NADH-cytochrome b5 reductase 1	1.66
KIF1A	Kinesin-like protein KIF1A	1.66
ENTPD8	Ectonucleoside triphosphate diphosphohydrolase 8	1.66
DLGAP4	Disks large-associated protein 4	1.66
IVD	Isovaleryl-CoA dehydrogenase, mitochondrial	1.66
MRPS18C	28S ribosomal protein S18c, mitochondrial	1.66
CTSD	Cathepsin D	1.66
HIBCH	3-hydroxyisobutyryl-CoA hydrolase, mitochondrial	1.66
HS1BP3	HCLS1-binding protein 3	1.66
MISP	Mitotic interactor and substrate of PLK1	1.66
ANXA2	Annexin A2	1.65
CD44	CD44 antigen	1.65
MSRB2	Methionine-R-sulfoxide reductase B2, mitochondrial	1.65
GLB1	Beta-galactosidase	1.64
CPD	Carboxypeptidase D	1.64

TACSTD2	Tumor-associated calcium signal transducer 2	1.64
COMTD1	Catechol O-methyltransferase domain-containing protein 1	1.64
RIN1	Ras and Rab interactor 1	1.63
CMAS	N-acylneuraminate cytidylyltransferase	1.63
NQO1	NAD(P)H dehydrogenase [quinone] 1	1.63
ERLEC1	Endoplasmic reticulum lectin 1	1.63
CDS2	Phosphatidate cytidylyltransferase 2	1.63
GLUD2	Glutamate dehydrogenase 2, mitochondrial	1.62
VDAC1	Voltage-dependent anion-selective channel protein 1	1.61
TTC19	Tetratricopeptide repeat protein 19, mitochondrial	1.61
SEMA3C	Semaphorin-3C	1.61
LRSAM1	E3 ubiquitin-protein ligase LRSAM1	1.60
ACOT13	Acyl-coenzyme A thioesterase 13	1.60
LXN	Latexin	1.60
GSN	Gelsolin	1.60
CHP1	Calcineurin B homologous protein 1	1.60
GALNT2	N-acetylgalactosaminyltransferase 2	1.60
RARS2	Arginine-tRNA ligase, mitochondrial	1.60
PACS1	Phosphofurin acidic cluster sorting protein 1	1.60
RMDN3	Regulator of microtubule dynamics protein 3	1.60
PANK4	Pantothenate kinase 4	1.59
KTN1	Kinectin	1.59
CTSB	Cathepsin B	1.58
BCKDHA	2-oxoisovalerate dehydrogenase subunit alpha, mitochondrial	1.58
EBAG9	Receptor-binding cancer antigen expressed on SiSo cells	1.58
TMEM214	Transmembrane protein 214	1.58
UQCC2	Ubiquinol-cytochrome-c reductase complex assembly factor 2, mitochondrial	1.58
TM9SF4	Transmembrane 9 superfamily member 4	1.58
HDHD2	Haloacid dehalogenase-like hydrolase domain-containing protein 2	1.58
EPHX1	Epoxide hydrolase 1	1.58
TMF1	TATA element modulatory factor	1.58
CDIPT	CDP-diacylglycerol--inositol 3-phosphatidyltransferase	1.57

CD81	CD81 antigen	1.57
SRXN1	Sulfiredoxin-1	1.57
ME1	NADP-dependent malic enzyme	1.57
ACOT8	Acyl-coenzyme A thioesterase 8, peroxisomal	1.57
SMDT1	Essential MCU regulator, mitochondrial	1.56
ALG1	Chitobiosyldiphosphodolichol beta-mannosyltransferase	1.56
DNAJC5	DnaJ homolog subfamily C member 5	1.55
DBT	Lipoamide acyltransferase component of branched-chain alpha-keto acid dehydrogenase complex, mitochondrial	1.55
LAMTOR2	Ragulator complex protein LAMTOR2	1.54
TIGAR	Fructose-2,6-bisphosphatase TIGAR	1.54
IDUA	Alpha-L-iduronidase	1.54
TMEM87B	Transmembrane protein 87B	1.54
TNKS1BP1	182 kDa tankyrase-1-binding protein	1.54
MIA3	Transport and Golgi organization protein 1 homolog	1.54
TXNRD1	Thioredoxin reductase 1, cytoplasmic	1.54
MYOF	Myoferlin	1.54
RABEP2	Rab GTPase-binding effector protein 2	1.53
GLUD1	Glutamate dehydrogenase 1, mitochondrial	1.53
PDF	Peptide deformylase, mitochondrial	1.53
TAPBP	Tapasin	1.53
NDUFS7	NADH dehydrogenase [ubiquinone] iron-sulfur protein 7, mitochondrial	1.53
ATP2C1	Calcium-transporting ATPase type 2C member 1	1.53
ANK3	Ankyrin-3	1.53
ABHD11	Protein ABHD11	1.53
AGO3	Protein argonaute-3	1.53
S100A16	Protein S100-A16	1.53
TM7SF2	Delta(14)-sterol reductase	1.53
MRPL21	39S ribosomal protein L21, mitochondrial	1.53
RAB9A	Ras-related protein Rab-9A	1.53
TOM1	Target of Myb protein 1	1.53
C21orf33	ES1 protein homolog, mitochondrial	1.52

SURF1	Surfeit locus 1 (cytochrome c oxidase assembly protein), mitochondrial	1.52
NAMPT	Nicotinamide phosphoribosyltransferase	1.51
METTL7B	Methyltransferase-like protein 7B	1.51
CTSA	Cathepsin A	1.51
TTC37	Tetratricopeptide repeat protein 37	1.51
RIDA	2-iminobutanoate/2-iminopropanoate deaminase	1.50
ARPC1A	Actin-related protein 2/3 complex subunit 1A	1.50
OS9	Protein OS-9	1.50
FUCA1	Tissue alpha-L-fucosidase	1.50

Table S2: Mitochondrial-related Proteins Up-regulated in e-CSCs, derived from MCF7 3D-Spheroids.

Symbol	Gene Description	Fold-Change (Up-regulation)
GLRX	Glutaredoxin-1	10.79
ALDH3A1	Aldehyde dehydrogenase, dimeric NADP-preferring	10.24
QPRT	Nicotinate-nucleotide pyrophosphorylase [carboxylating]	3.72
MIC13	MICOS complex subunit MIC13, mitochondrial	2.36
OXSM	3-oxoacyl-[acyl-carrier-protein] synthase, mitochondrial	2.22
FECH	Ferrochelatase, mitochondrial	2.16
MCU	Calcium uniporter protein, mitochondrial	2.13
GCLC	Glutamate--cysteine ligase catalytic subunit	2.01
NQO2	Ribosyldihydronicotinamide dehydrogenase [quinone]	1.90
CMC1	COX assembly mitochondrial protein homolog	1.87
ISCU	Iron-sulfur cluster assembly enzyme ISCU, mitochondrial	1.83
ABAT	4-aminobutyrate aminotransferase, mitochondrial	1.81
ABCB6	ATP-binding cassette sub-family B member 6, mitochondrial	1.80
ACADSB	Short/branched chain specific acyl-CoA dehydrogenase, mitochondrial	1.79
COQ9	Ubiquinone biosynthesis protein COQ9, mitochondrial	1.77
ALDH5A1	Succinate-semialdehyde dehydrogenase, mitochondrial	1.76
CPT2	Carnitine O-palmitoyltransferase 2, mitochondrial	1.76
HMGCL	Hydroxymethylglutaryl-CoA lyase, mitochondrial	1.75
FDXR	NADPH:adrenodoxin oxidoreductase, mitochondrial	1.75
CYCS	Cytochrome c	1.74
SQOR	Sulfide:quinone oxidoreductase, mitochondrial	1.73
HMOX1	Heme oxygenase 1	1.69
MLYCD	Malonyl-CoA decarboxylase, mitochondrial	1.67
ENDOG	Endonuclease G, mitochondrial	1.67
IVD	Isovaleryl-CoA dehydrogenase, mitochondrial	1.66
MRPS18C	28S ribosomal protein S18c, mitochondrial	1.66
HIBCH	3-hydroxyisobutyryl-CoA hydrolase, mitochondrial	1.66
MSRB2	Methionine-R-sulfoxide reductase B2, mitochondrial	1.64

NQO1	NAD(P)H dehydrogenase [quinone] 1	1.63
GLUD2	Glutamate dehydrogenase 2, mitochondrial	1.62
VDAC1	Voltage-dependent anion-selective channel protein 1	1.61
TTC19	Tetratricopeptide repeat protein 19, mitochondrial	1.61
ACOT13	Acyl-coenzyme A thioesterase 13, mitochondrial	1.60
RARS2	Arginine-tRNA ligase, mitochondrial	1.60
BCKDHA	2-oxoisovalerate dehydrogenase subunit alpha, mitochondrial	1.58
UQCC2	Ubiquinol-cytochrome-c reductase complex assembly factor 2, mitochondrial	1.58
ME1	NADP-dependent malic enzyme	1.57
SMDT1	Essential MCU regulator, mitochondrial	1.56
DNAJC5	DnaJ homolog subfamily C member 5	1.55
DBT	Lipoamide acyltransferase/branched-chain α-keto dehydrogenase, mitochondrial	1.55
TIGAR	Fructose-2,6-bisphosphatase TIGAR	1.54
GLUD1	Glutamate dehydrogenase 1, mitochondrial	1.53
PDF	Peptide deformylase, mitochondrial	1.53
NDUFS7	NADH dehydrogenase [ubiquinone] iron-sulfur protein 7, mitochondrial	1.53
MRPL21	39S ribosomal protein L21, mitochondrial	1.53
C21orf33	ES1 protein homolog, mitochondrial	1.52
SURF1	Surfeit locus 1 (cytochrome c oxidase assembly protein), mitochondrial	1.52
NAMPT	Nicotinamide phosphoribosyltransferase	1.51

Table S3: Functional Markers of the e-CSC Phenotype (from MCF7 3D-Spheroids).

Symbol	Gene Description	Fold-Change (Up-regulation)
**Senescence Markers**		
CDKN1A	Cyclin-dependent kinase inhibitor 1 (p21-WAF/CDK-inhibitor)	17.22
GLB1	Beta-galactosidase	1.64
**Anti-Oxidant Response to ROS/Oxidative Stress**		
GLRX	Glutaredoxin-1	10.79
GCLC	Glutamate--cysteine ligase catalytic subunit	2.01
NQO2	Ribosyldihydronicotinamide dehydrogenase [quinone]	1.90
MGST1	Microsomal glutathione S-transferase 1	1.88
MGST3	Microsomal glutathione S-transferase 3	1.84
SPATA20	Spermatogenesis-associated protein 20 (thioredoxin-like)	1.77
GSTK1	Glutathione S-transferase kappa 1	1.75
NQO1	NAD(P)H dehydrogenase [quinone] 1	1.63
**Stemness & Drug-Resistance/Radio-Resistance**		
BCAS1	Breast carcinoma-amplified sequence 1	119.37
ALDH3A1	Aldehyde dehydrogenase, dimeric NADP-preferring	10.24
CEACAM6	Carcinoembryonic antigen-related cell adhesion molecule 6	9.66
CEACAM5	Carcinoembryonic antigen-related cell adhesion molecule 5	3.38
LAMA5	Laminin subunit alpha-5	2.72
ALDH5A1	Succinate-semialdehyde dehydrogenase, mitochondrial	1.76
CD44	CD44 antigen	1.65
**Cytoskeletal Proteins (indicative of an EMT in CSCs)**		
TUBA4A	Tubulin alpha-4A chain	2.60
STOM	Erythrocyte band 7 integral membrane protein	2.18
MAPRE3	Microtubule-associated protein RP/EB family member 3	1.74

KIF1A	Kinesin-like protein KIF1A	1.66
RMDN3	Regulator of microtubule dynamics protein 3	1.60
GSN	Gelsolin	1.60
MYOF	Myoferlin	1.54
ANK3	Ankyrin-3	1.53
ARPC1A	Actin-related protein 2/3 complex subunit 1A	1.50
**Spindle Orientation and Mitotic Progression**		
MISP	Mitotic interactor and substrate of PLK1	1.66
**Mitochondrial Biogenesis**		
GLRX	Glutaredoxin-1	10.79
MIC13	MICOS complex subunit MIC13, mitochondrial	2.36
OXSM	3-oxoacyl-[acyl-carrier-protein] synthase, mitochondrial	2.22
FECH	Ferrochelatase, mitochondrial	2.16
CMC1	COX assembly mitochondrial protein homolog	1.87
ISCU	Iron-sulfur cluster assembly enzyme ISCU, mitochondrial	1.83
COQ9	Ubiquinone biosynthesis protein COQ9, mitochondrial	1.77
HMOX1	Heme oxygenase 1	1.69
UQCC2	Ubiquinol-cytochrome-c reductase complex assembly factor 2, mitochondrial	1.58
MRPS18C	28S ribosomal protein S18c, mitochondrial	1.66
RARS2	Arginine-tRNA ligase, mitochondrial	1.60
MRPL21	39S ribosomal protein L21, mitochondrial	1.53
PDF	Peptide deformylase, mitochondrial	1.53
**Glutamine/Asparagine Metabolism**		
AGA	N(4)-(beta-N-acetylglucosaminyl)-L-asparaginase	2.30
GLUD2	Glutamate dehydrogenase 2, mitochondrial	1.62
GLUD1	Glutamate dehydrogenase 1, mitochondrial	1.53

NADH /NADPH: Synthesis & Salvage Pathway

ALDH3A1	Aldehyde dehydrogenase, dimeric NADP-preferring	10.24
QPRT	Nicotinate-nucleotide pyrophosphorylase [carboxylating]	3.72
RRM2	Ribonucleoside-diphosphate reductase subunit M2	2.34
ALDH5A1	Succinate-semialdehyde dehydrogenase, mitochondrial	1.76
FDXR	NADPH:adrenodoxin oxidoreductase, mitochondrial	1.75
RRM2B	Ribonucleoside-diphosphate reductase subunit M2 B	1.68
ME1	NADP-dependent malic enzyme	1.57
TIGAR	Fructose-2,6-bisphosphatase TIGAR	1.54
TNKS1BP1	182 kDa tankyrase-1-binding protein	1.54
NDUFS7	NADH dehydrogenase [ubiquinone] iron-sulfur protein 7, mitochondrial	1.53
NAMPT	Nicotinamide phosphoribosyltransferase	1.51

Flavin-containing Enzymes

CYP1A1	Cytochrome P450 1A1	6.60
MAOA	Amine oxidase [flavin-containing] A	4.73
MAOB	Amine oxidase [flavin-containing] B	2.45
GFER	FAD-linked sulfhydryl oxidase ALR	1.80
CYB5R1	NADH-cytochrome b5 reductase 1	1.66
TXNRD1	Thioredoxin reductase 1, cytoplasmic (Glutaredoxin activity; flavin-dependent)	1.54

Epithelial Markers

KRT10	Keratin, type I cytoskeletal 10	4.59
DPP7	Dipeptidyl peptidase 2	2.43
MUC5B	Mucin-5B	1.98

Cell Surface Markers

GM2A	Ganglioside GM2 activator	2.36
CD59	CD59 glycoprotein	1.80
ENTPD8	Ectonucleoside triphosphate diphosphohydrolase 8	1.66
CD81	CD81 antigen	1.57

S100 Proteins

S100P	Protein S100-P	2.11
S100A10	Protein S100-A10	1.96
S100A16	Protein S100-A16	1.53

Autophagy/Lysosomes

CHMP6	Charged multivesicular body protein 6	2.62
SERPINA3	Alpha-1-antichymotrypsin	2.33
CTSH	Cathepsin H	1.98
GNS	N-acetylglucosamine-6-sulfatase	1.97
GAA	Lysosomal alpha-glucosidase	1.83
GALNS	N-acetylgalactosamine-6-sulfatase	1.82
ATP6V0A1	V-type proton ATPase 116 kDa subunit a isoform 1	1.71
CTSD	Cathepsin D	1.66
CPD	Carboxypeptidase D	1.64
GALNT2	N-acetylgalactosaminyltransferase 2	1.60
CTSB	Cathepsin B	1.58
CTSA	Cathepsin A	1.51

Peroxisomes

PEX14	Peroxisomal membrane protein PEX14	1.91
DECR2	Peroxisomal 2,4-dienoyl-CoA reductase	1.81
PEX11B	Peroxisomal membrane protein PEX11B	1.76
ACOT8	Acyl-coenzyme A thioesterase 8, peroxisomal	1.57

RABs

RAB27B	Ras-related protein Rab-27B	2.69
RAB27A	Ras-related protein Rab-27A	2.54
RAB24	Ras-related protein Rab-24	1.87
RIN1	Ras and Rab interactor 1	1.63
RABEP2	Rab GTPase-binding effector protein 2	1.53
RAB9A	Ras-related protein Rab-9A	1.53

**Annexins and PARP**		
ANXA1	Annexin A1	2.87
PARP4	Poly [ADP-ribose] polymerase 4	2.55
ANXA2	Annexin A2	1.65
**Calcium/Calmodulin**		
CIB1	Calcium and integrin-binding protein 1	2.90
MCU	Calcium uniporter protein, mitochondrial	2.13
CAPS	Calcyphosin	1.79
CAMK2D	Calcium/calmodulin-dependent protein kinase, type II subunit delta	1.70
CALML5	Calmodulin-like protein 5	1.69
TACSTD2	Tumor-associated calcium signal transducer 2	1.64
CHP1	Calcineurin B homologous protein 1	1.60
SMDT1	Essential MCU regulator, mitochondrial	1.56
ATP2C1	Calcium-transporting ATPase type 2C member 1	1.53

Table S4: e-CSC Biomarkers are Transcriptionally Up-regulated in Patient-derived Breast Cancer Cells In Vivo.

Symbol	Gene Description	Fold-Change	P-value
TSPAN31	Tetraspanin-31	4.72	8.45E-06
CDS2	Phosphatidate cytidylyltransferase 2	4.71	8.73E-06
PEX11B	Peroxisomal membrane protein PEX11B	4.69	9.58E-06
RAB9A	Ras-related protein Rab-9A	4.47	2.02E-05
TACSTD2	Tumor-associated calcium signal transducer 2	4.41	2.47E-05
**GLUD1**	**Glutamate dehydrogenase 1, mitochondrial**	**4.38**	**2.76E-05**
**MSRB2**	**Methionine-R-sulfoxide reductase B2, mitochondrial**	**4.31**	**3.49E-05**
**SURF1**	**Surfeit locus 1 (cytochrome c oxidase assembly protein), mitochondrial**	**4.16**	**5.66E-05**
PON2	Serum paraoxonase/arylesterase 2	4.01	9.25E-05
CYB5R1	NADH-cytochrome b5 reductase 1	3.94	1.18E-04
ANK3	Ankyrin-3	3.81	1.77E-04
ASAH1	Acid ceramidase	3.80	1.83E-04
CD59	CD59 glycoprotein	3.60	3.47E-04
**OXSM**	**3-oxoacyl-[acyl-carrier-protein] synthase, mitochondrial**	**3.49**	**4.82E-04**
**NQO1**	**NAD(P)H dehydrogenase [quinone] 1**	**3.49**	**4.81E-04**
SEMA3C	Semaphorin-3C	3.49	4.92E-04
CD44	CD44 antigen	3.44	5.69E-04
**ALDH5A1**	**Succinate-semialdehyde dehydrogenase, mitochondrial**	**3.43**	**5.75E-04**
AGA	N(4)-(beta-N-acetylglucosaminyl)-L-asparaginase	3.40	6.30E-04
GSTK1	Glutathione S-transferase kappa 1	3.39	6.59E-04
KTN1	Kinectin	3.36	7.16E-04
**FECH**	**Ferrochelatase, mitochondrial**	**3.36**	**7.20E-04**
**C21orf33**	**ES1 protein homolog, mitochondrial**	**3.31**	**8.40E-04**
MPV17	Protein Mpv17	3.27	9.44E-04
TMEM214	Transmembrane protein 214	3.12	1.44E-03
NEBL	Nebulette	3.09	1.59E-03
CDIPT	CDP-diacylglycerol--inositol 3-phosphatidyltransferase	3.06	1.74E-03

**CPT2**	**Carnitine O-palmitoyltransferase 2, mitochondrial**	**3.02**	**1.94E-03**
ATP2C1	Calcium-transporting ATPase type 2C member 1	3.01	1.96E-03
SERPINA3	Alpha-1-antichymotrypsin	2.99	2.11E-03
**CYCS**	**Cytochrome c**	**2.92**	**2.52E-03**
**TTC19**	**Tetratricopeptide repeat protein 19, mitochondrial**	**2.85**	**3.06E-03**
SELENBP1	Methanethiol oxidase	2.84	3.22E-03
MIA3	Transport and Golgi organization protein 1 homolog	2.76	3.98E-03
OS9	Protein OS-9; amplified in osteosarcoma	2.76	3.99E-03
ANXA2	Annexin A2	2.73	4.30E-03
SULT1A1	Sulfotransferase 1A1	2.72	4.34E-03
MYOF	Myoferlin	2.67	5.00E-03
CAPN2	Calpain-2 catalytic subunit	2.64	5.42E-03
**VDAC1**	**Voltage-dependent anion-selective channel protein 1, mitochondrial**	**2.64**	**5.35E-03**
TXNRD1	Thioredoxin reductase 1, cytoplasmic	2.64	5.36E-03
EPS8L1	Epidermal growth factor receptor kinase substrate 8-like protein 1	2.57	6.54E-03
**PDF**	**Peptide deformylase, mitochondrial**	**2.56**	**6.71E-03**
CTSH	Cathepsin H	2.54	7.07E-03
KRT10	Keratin, type I cytoskeletal 10	2.53	7.19E-03
GLB1	Beta-galactosidase	2.53	7.20E-03
GM2A	Ganglioside GM2 activator	2.42	9.42E-03
RRM2	Ribonucleoside-diphosphate reductase subunit M2	2.40	9.93E-03
RETSAT	All-trans-retinol 13,14-reductase	2.39	1.03E-02
RNASET2	Ribonuclease T2	2.36	1.10E-02
**ENDOG**	**Endonuclease G, mitochondrial**	**2.32**	**1.22E-02**
NAMPT	Nicotinamide phosphoribosyltransferase	2.19	1.66E-02
SPATA20	Spermatogenesis-associated protein 20	2.16	1.77E-02
SLC22A18	Solute carrier family 22 member 18	2.14	1.86E-02
**ABAT**	**4-aminobutyrate aminotransferase, mitochondrial**	**2.08**	**2.14E-02**
TAPBP	Tapasin	2.08	2.13E-02
CIB1	Calcium and integrin-binding protein 1	2.04	2.34E-02
**HMGCL**	**Hydroxymethylglutaryl-CoA lyase, mitochondrial**	**2.03**	**2.38E-02**
FAM8A1	Protein FAM8A1	2.02	2.40E-02

**GCLC**	**Glutamate--cysteine ligase catalytic subunit**	**2.01**	**2.49E-02**
ACAA1	3-ketoacyl-CoA thiolase, peroxisomal	2.00	2.53E-02
**GLRX**	**Glutaredoxin-1**	**1.92**	**3.01E-02**
**ISCU**	**Iron-sulfur cluster assembly enzyme ISCU, mitochondrial**	**1.92**	**3.02E-02**
TMF1	TATA element modulatory factor	1.88	3.25E-02
CD81	CD81 antigen	1.87	3.34E-02
**NQO2**	**Ribosyldihydronicotinamide dehydrogenase [quinone]**	**1.79**	**3.98E-02**
MAOB	Amine oxidase [flavin-containing] B	1.74	4.41E-02
CEACAM6	Carcinoembryonic antigen-related cell adhesion molecule 6	1.70	4.71E-02
SLC9A1	Sodium/hydrogen exchanger 1	1.68	4.97E-02

Markers highlighted in BOLD are Mitochondrial-related proteins.

Table S5: Tumor Recurrence (RFS): Predicting Tamoxifen-resistance in ER(+) Breast Cancer Patients.

Gene Probe	Gene Symbol	HR (Hazard-Ratio)	Log-Rank Test
201468_s_at	NQO1	2.47	0.0023
203608_at	ALDH5A1	2.21	0.01
201266_at	TXNR	2.17	0.0062
201890_at	RRM2	2.54	0.00089
**Combined Signature (RFS)**		**3.89**	**4.1e-05**

(RFS, recurrence-free survival)

Table S6: Distant Metastasis (DMFS): Predicting Tamoxifen-resistance in ER(+) Breast Cancer Patients.

Gene Probe	Gene Symbol	HR (Hazard-Ratio)	Log-Rank Test
201468_s_at	NQO1	1.73	0.1
203608_at	ALDH5A1	2.86	0.0034
201266_at	TXNR	3.64	0.00035
201890_at	RRM2	3.02	0.00092

(DMFS, distant metastasis-free survival)
