# Supplementary material for: “Energetic” Cancer Stem Cells (e-CSCs): A New Hyper-Metabolic and Proliferative Tumor Cell Phenotype, Driven by Mitochondrial Energy
Source: Front Oncol. 2019 Feb 5;8:677. doi: 10.3389/fonc.2018.00677 (PMC6370664; doi:10.3389/fonc.2018.00677)
Supplement: Supplementary file 2 [file Data_Sheet_2.PDF]

Supplemental Material (Figures S1-S4)

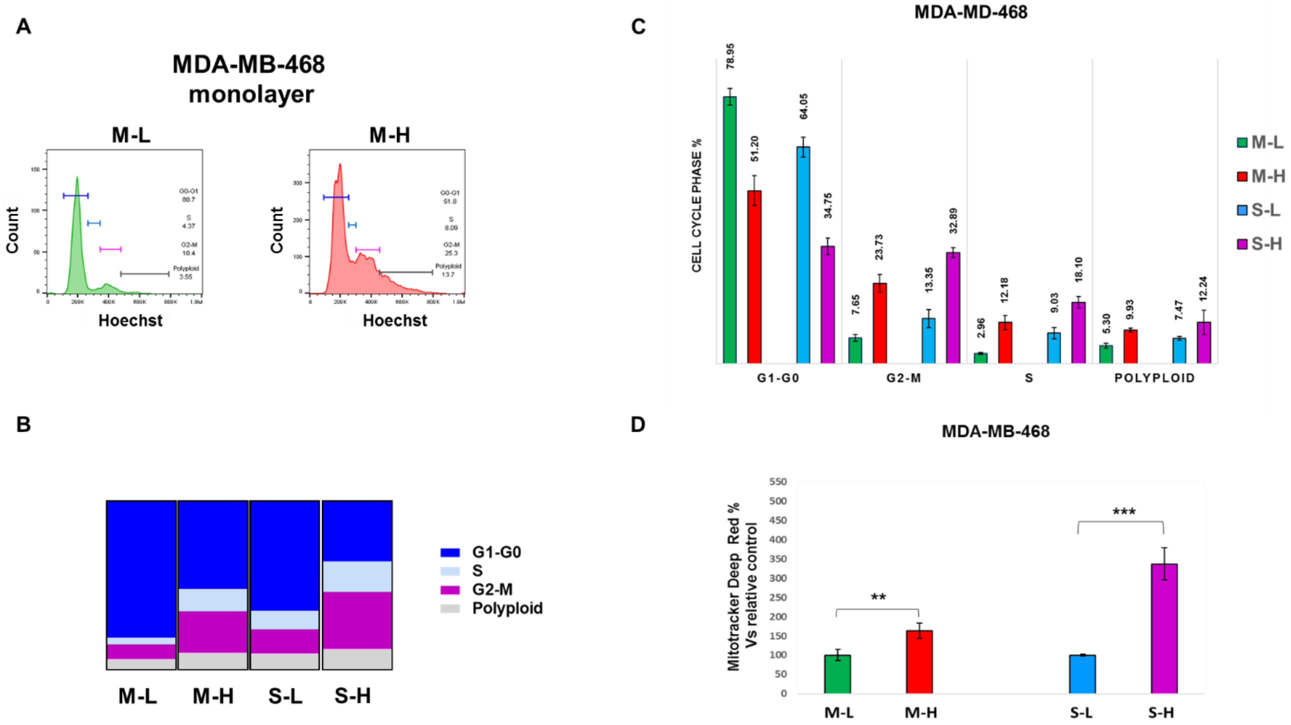

Figure S1.

MDA-MB-468 e-CSCs demonstrate increased cell cycle progression and increased mitochondrial mass. A) Representative images of cell cycle analysis. B & C) Analysis of cell cycle progression. See also Table 2. D) Analysis of mitochondrial mass with MitoTracker vital staining. Note that the S-H cell sub-population derived from MDA-MB-468 cells shows the largest increases in cell cycle progression and mitochondrial mass. Virtually identical results were obtained with MCF7 cells. Compare with data presented in Figures 5 & 6.

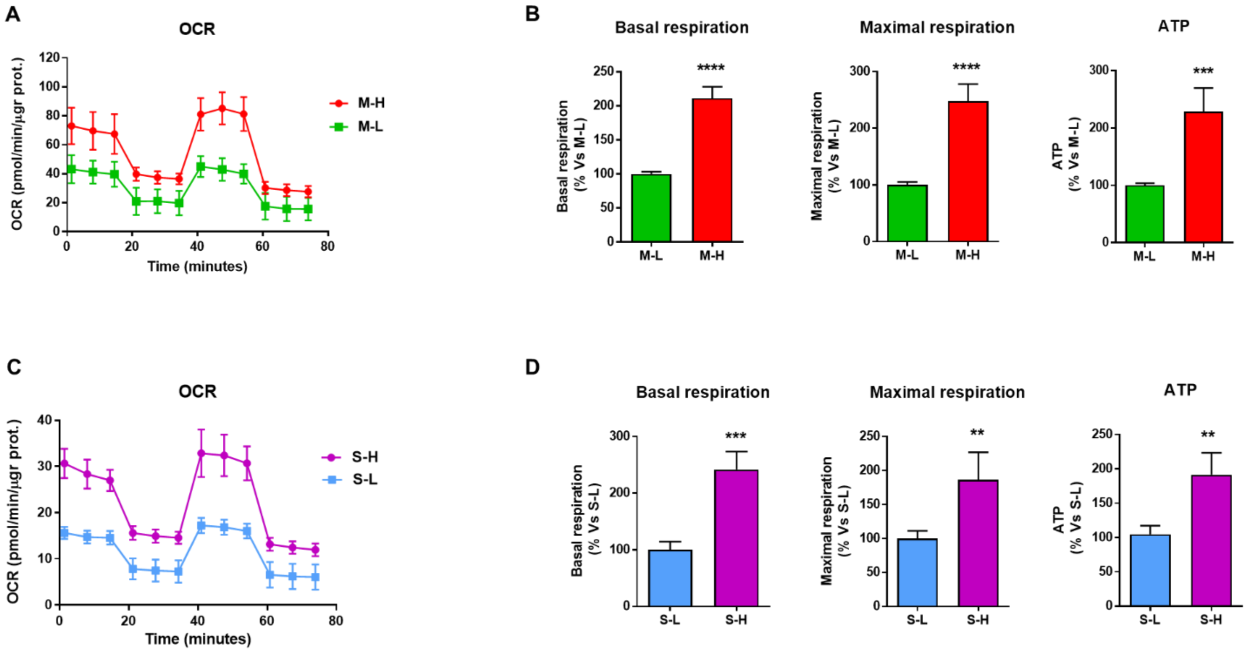

Figure S2.

MDA-MB-468 e-CSCs have increased oxidative mitochondrial metabolism. The oxygen consumption rate (OCR) was measured, using the Seahorse XFe96 metabolic-flux analyzer. Note that high OCR in MDA-MB-468 cells directly correlates with high-flavin content. For example, M-H cells (from 2D-monolayers) and S-H cells (from 3D-spheroids) have the highest levels of OCR, as compared to the M-L and S-L sub-populations. A, B) OCR for M-L vs. M-H sub-populations; C, D) OCR for S-L vs. S-H sub-populations.

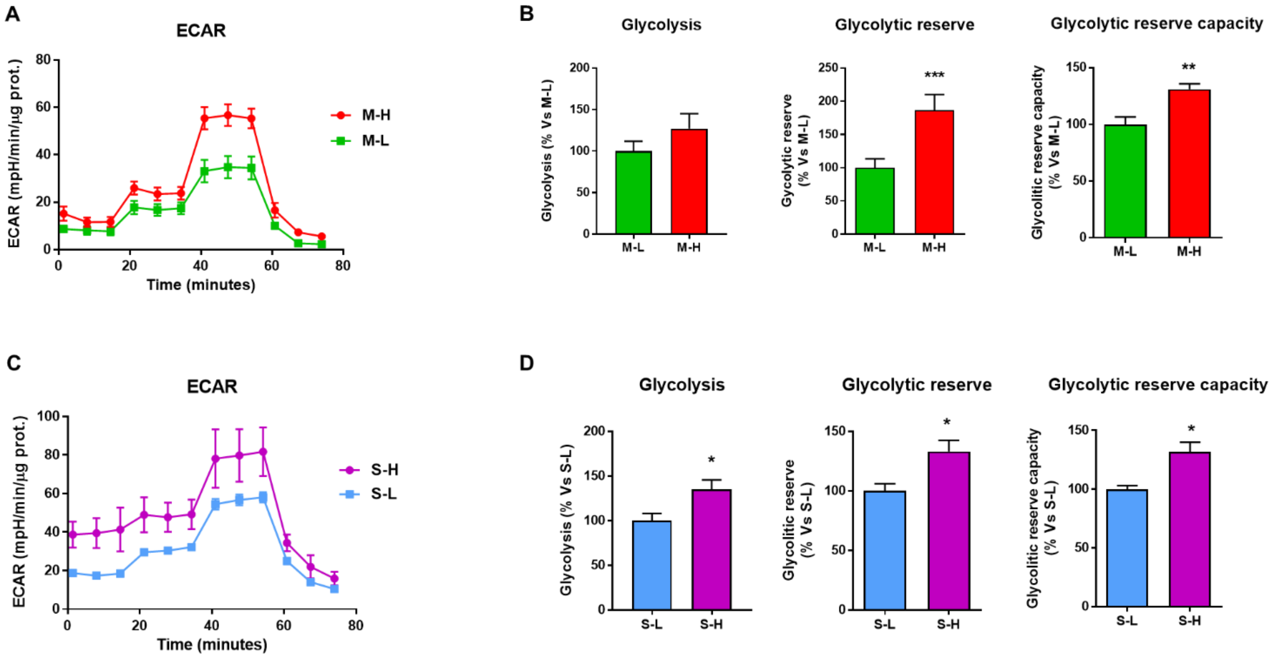

Figure S3.

MDA-MB-468 e-CSCs have elevated levels of aerobic glycolysis. The extracellular acidification rate (ECAR) was measured, using the Seahorse XFe96 metabolic-flux analyzer. Note that high ECAR in MDA-MD-468 cells directly correlates with high-flavin content. For example, M-H cells (from 2D-monolayers) and S-H cells (from 3D-spheroids) have the highest levels of ECAR, as compared to the M-L and S-L sub-populations. A, B) ECAR for M-L vs. M-H sub-populations; C, D) ECAR for S-L vs. S-H sub-populations

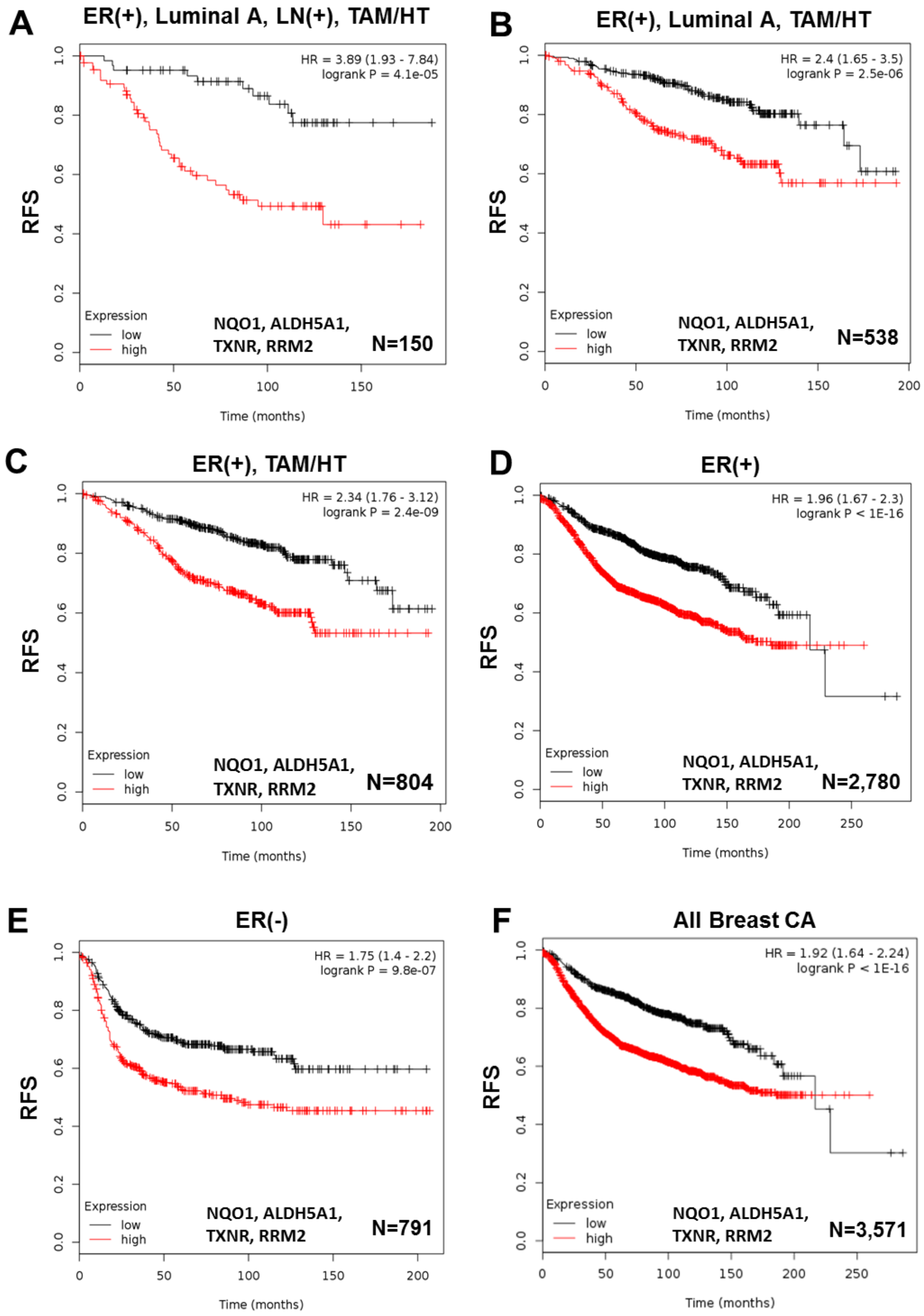
A
ER(+), Luminal A, LN(+), TAM/HT
HR = 3.89 (1.93 - 7.84)
logrank P = 4.1e-05
RFS
Expression
low
high
NQO1, ALDH5A1,
TXNR, RRM2
N=150
Time (months)
B
ER(+), Luminal A, TAM/HT
HR = 2.4 (1.65 - 3.5)
logrank P = 2.5e-06
RFS
Expression
low
high
NQO1, ALDH5A1,
TXNR, RRM2
N=538
Time (months)
C
ER(+), TAM/HT
HR = 2.34 (1.76 - 3.12)
logrank P = 2.4e-09
RFS
Expression
low
high
NQO1, ALDH5A1,
TXNR, RRM2
N=804
Time (months)
D
ER(+)
HR = 1.96 (1.67 - 2.3)
logrank P < 1E-16
RFS
Expression
low
high
NQO1, ALDH5A1,
TXNR, RRM2
N=2,780
Time (months)
E
ER(-)
HR = 1.75 (1.4 - 2.2)
logrank P = 9.8e-07
RFS
Expression
low
high
NQO1, ALDH5A1,
TXNR, RRM2
N=791
Time (months)
F
All Breast CA
HR = 1.92 (1.64 - 2.24)
logrank P < 1E-16
RFS
Expression
low
high
NQO1, ALDH5A1,
TXNR, RRM2
N=3,571
Time (months)

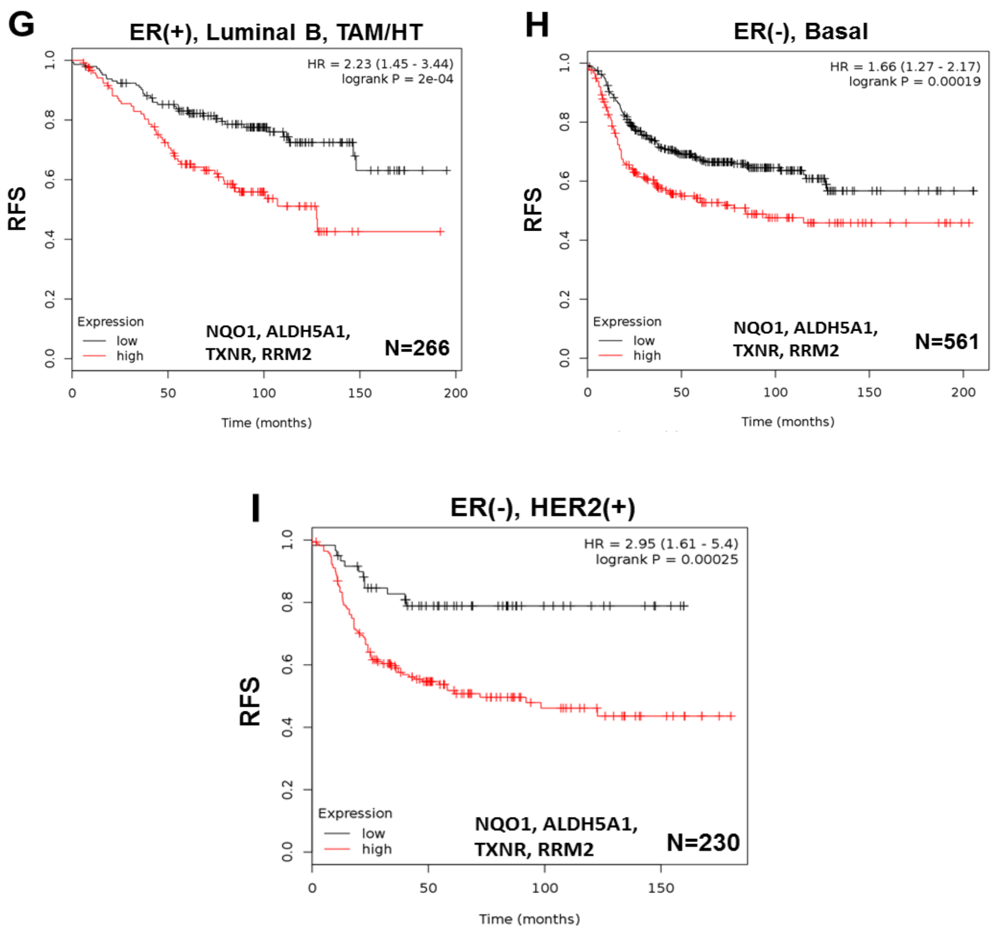

Figure S4.

K-M analysis of human breast cancer patient sub-types, using an anti-oxidant signature derived from e-CSCs. The K-M curves directly show that the anti-oxidant signature from e-CSCs effectively predicts tumor recurrence in all of the breast cancer sub-types tested. The patient groups examined were as follows: A) ER(+), Luminal A sub-type, with Lymph-Node metastasis (LN(+)) at diagnosis, and treated with hormonal therapy (TAM/HT) (**N=150**); B) ER(+), Luminal A sub-type, and treated with hormonal therapy (TAM/HT) (**N=538**); C) ER(+) and treated with hormonal therapy (TAM/HT) (**N=804**); D) All ER(+) (**N=2,780**); E) All ER(-) (**N=791**); F) All Breast Cancer (**N=3,571**); G) ER(+), with the Luminal B sub-type (**N=266**); H) ER(-), with the Basal sub-type (**N=561**); and I) ER(-) and HER2(+) (**N=230**).

RFS, recurrence-free survival.
